# The ribonuclease PNPase is a key regulator of biofilm formation in *Listeria monocytogenes* and affects invasion of host cells

**DOI:** 10.1038/s41522-023-00397-1

**Published:** 2023-06-07

**Authors:** Ana Patrícia Quendera, Sandra Nunes Pinto, Vânia Pobre, Wilson Antunes, Vasco D. B. Bonifácio, Cecília Maria Arraiano, José Marques Andrade

**Affiliations:** 1 Instituto de Tecnologia Química e Biológica António Xavier, Universidade Nova de Lisboa (ITQB NOVA), Avenida da República, 2780-901 Oeiras, Portugal; 2 Institute for Bioengineering and Biosciences (IBB) and Associate Laboratory—Institute for Health and Bioeconomy (i4HB), Instituto Superior Técnico, Universidade de Lisboa, Av. Rovisco Pais, 1049-001 Lisboa, Portugal; 3 Laboratório de Imagem, Nanomorfologia e Espectroscopia de Raios-X (Linx) da Unidade Militar Laboratorial de Defesa Biológica e Química (UMLDBQ), Instituto Universitário Militar, Centro de Investigação, Inovação e Desenvolvimento da Academia Militar, Av. Dr Alfredo Bensaúde, 1100-471 Lisboa, Portugal; 4 Bioengineering Department, Instituto Superior Técnico, Universidade de Lisboa, Av. Rovisco Pais, 1049-001 Lisboa, Portugal

**Keywords:** Biofilms, Pathogens, Microbial genetics

## Abstract

Biofilms provide an environment that protects microorganisms from external stresses such as nutrient deprivation, antibiotic treatments, and immune defences, thereby creating favorable conditions for bacterial survival and pathogenesis. Here we show that the RNA-binding protein and ribonuclease polynucleotide phosphorylase (PNPase) is a positive regulator of biofilm formation in the human pathogen *Listeria monocytogenes*, a major responsible for food contamination in food-processing environments. The PNPase mutant strain produces less biofilm biomass and exhibits an altered biofilm morphology that is more susceptible to antibiotic treatment. Through biochemical assays and microscopical analysis, we demonstrate that PNPase is a previously unrecognized regulator of the composition of the biofilm extracellular matrix, greatly affecting the levels of proteins, extracellular DNA, and sugars. Noteworthy, we have adapted the use of the fluorescent complex ruthenium red-phenanthroline for the detection of polysaccharides in *Listeria* biofilms. Transcriptomic analysis of wild-type and PNPase mutant biofilms reveals that PNPase impacts many regulatory pathways associated with biofilm formation, particularly by affecting the expression of genes involved in the metabolism of carbohydrates (e.g., *lmo0096* and *lmo0783*, encoding PTS components), of amino acids (e.g., *lmo1984* and *lmo2006*, encoding biosynthetic enzymes) and in the Agr quorum sensing-like system (*lmo0048-49*). Moreover, we show that PNPase affects mRNA levels of the master regulator of virulence PrfA and PrfA-regulated genes, and these results could help to explain the reduced bacterial internalization in human cells of the Δ*pnpA* mutant. Overall, this work demonstrates that PNPase is an important post-transcriptional regulator for virulence and adaptation to the biofilm lifestyle of Gram-positive bacteria and highlights the expanding role of ribonucleases as critical players in pathogenicity.

## Introduction

Biofilms are the prevalent mode of growth for microorganisms in natural ecosystems. However, they have been an increasing medical concern given that 80% of microbial infections are associated with biofilms^1,2^ and contribute to the persistence of chronic infections^3^. Biofilms are defined as communities of microorganisms that are attached to biotic or abiotic surfaces and are enclosed in self-produced hydrated extracellular polymeric substances (EPS), also known as the biofilm matrix^4,5^. Polysaccharides, proteins, phospholipids, and nucleic acids are the main components of the biofilm extracellular matrix^6^. Sessile microorganisms are protected by the surrounding matrix and can endure external stresses like nutrient deprivation and desiccation much better than planktonic bacteria, as well as being much less susceptible to the action of antimicrobial agents and host’s immune defences^7^. The biofilm’s structure allows the accumulation of components of lysed cells and favors horizontal gene transfer between bacteria and cell-cell communication^6,8^.

Biofilm formation has been associated with virulence in several pathogenic bacteria by promoting infection. *Listeria monocytogenes* (*Listeria*) is a Gram-positive pathogenic bacterium that causes listeriosis, one of the most lethal foodborne infections in humans^9^. It can survive intracellularly, manipulate host’s cellular machineries, and escape from immune system^10^. *L. monocytogenes* endures adverse environmental conditions such as low temperatures, low pH, and high salt concentrations^11^. Moreover, it can adhere to several types of surfaces, persist in the form of biofilms in food-processing environments and contaminate food products, making this bacterium a major burden for the food industry^12–15^. When grown under static conditions, *L. monocytogenes* biofilms can either be in the form of a bacterial monolayer^16^, or form a honeycomb-like biofilm^17^. They can also form ball-shaped structures under continuous-flow conditions^18^.

Biofilm formation is a complex and multifactorial process. In *L. monocytogenes*, as in other bacterial pathogens, it has been shown that swimming ability^19^ and the Agr quorum sensing-like system are important at the early stages of biofilm formation, with the latter being involved in the regulation of proteins relevant for adhesion to surfaces and/or bacteria^20,21^. Moreover, both the transcriptional regulator of virulence PrfA and the stress response regulator σ^B^ are important for biofilm development in *Listeria*, namely by controlling the expression of genes such as *actA, inlA,* and *rmlA*^22–25^. For instance, it was shown that PrfA-regulated factor ActA is involved in bacterial aggregation, a key step in biofilm formation^24^. PrfA is responsible for the expression of several virulence factors, such as the surface proteins internalins, which are essential for invasion of mammalian cell lines^10,26^. In addition, other factors play a role in biofilm formation in pathogenic bacteria, such as sRNAs^27,28^ and c-di-GMP^29,30^.

Another factor found to regulate biofilm formation in other bacterial species is the RNA-binding protein polynucleotide phosphorylase (PNPase). It is a highly conserved 3’-5’ exoribonuclease that catalyzes the degradation and processing of RNA^31^ and has been implicated in virulence-related processes in different bacterial species^32–34^. We had previously found that PNPase is an important enzyme in *L. monocytogenes* in the processing and function of a CRISPR element^35^. In Gram-negative bacteria *Escherichia coli* K-12 and *Salmonella enterica* serovar Typhimurium, the inactivation of PNPase was shown to impair biofilm formation^36–38^. However, in *E. coli* C strain, the PNPase-deletion mutant showed increased biofilm formation^39^. Thus, the role of PNPase in biofilm formation remains to be fully explained, while it is still unknown if PNPase affects biofilm formation in Gram-positive bacteria.

In this work, we show that inactivation of PNPase in *L. monocytogenes* causes reduced cellular invasion and strong defects in biofilm production, greatly affecting the extracellular matrix composition. Also, RNA-sequencing analysis showed that PNPase is involved in the regulation of distinct pathways influencing biofilm formation, namely quorum sensing and metabolism of carbohydrates and amino acids. Hence, we present PNPase as a new biofilm regulator in Gram-positive pathogens.

## Results

### PNPase affects the internalization of *Listeria monocytogenes* in human cell lines

**Fig. 1:**
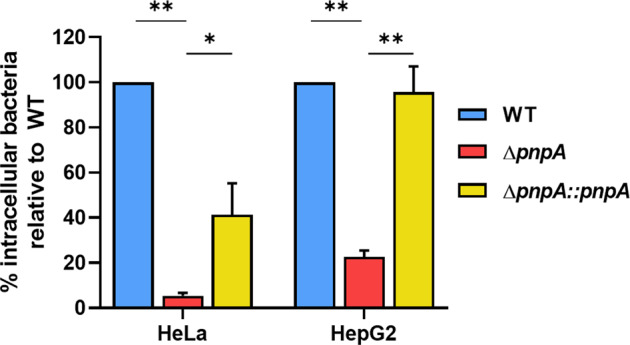
PNPase is important for *Listeria monocytogenes* invasion of human epithelial cell lines. Quantification of intracellular bacteria in HeLa and HepG2 cells at 2 hours post-infection, at MOI 50 for HeLa and MOI 40 for HepG2. Averaged replicate values were normalized to inoculum concentration (CFU/mL at the time of infection) and the transformed data expressed as the percentage of surviving bacteria relative to the wild-type. Data represent mean ± SD of three independent experiments. Significance was determined by an unpaired *t* test; *P < 0.05, **P < 0.01.

PNPase has been implicated in virulence-related processes in different bacterial species, even though its role is still not completely understood and may differ among different bacteria (reviewed in ref. ^31^). To determine if PNPase is important for *L. monocytogenes* virulence, we tested the ability of the wild-type (WT), the PNPase-deletion mutant (Δ*pnpA*) and the PNPase-complemented (Δ*pnpA::pnpA*) strains to invade host cells. For that, we used two well-established human epithelial cell lines: HeLa (isolated from cervical cancer and commonly used as a model of study) and HepG2 (hepatocytes-derived cells, as the liver is a preferred target for *Listeria* infection). Bacterial cultures were used to infect the human cells for one hour, followed by a one hour-treatment with gentamycin that kills extracellular bacteria^40^. Intracellular bacteria were recovered after host cell lysis, and viability was determined on BHI agar plates. Our results showed that PNPase was necessary for entry into both cell lines, as the Δ*pnpA* mutant was less invasive than the wild-type (decrease of ~95% in HeLa and 80% in HepG2) (Fig. 1). The complemented strain showed partial recovery in the infection of HeLa and full complementation in the infection of HepG2. These results demonstrate that PNPase is important for *L. monocytogenes* infection. This fact is in accordance with what has been described for other pathogenic bacteria^34^ and highlights a role for PNPase in bacterial virulence.

### Inactivation of PNPase leads to mutant phenotypes of *Listeria monocytogenes*

**Fig. 2:**
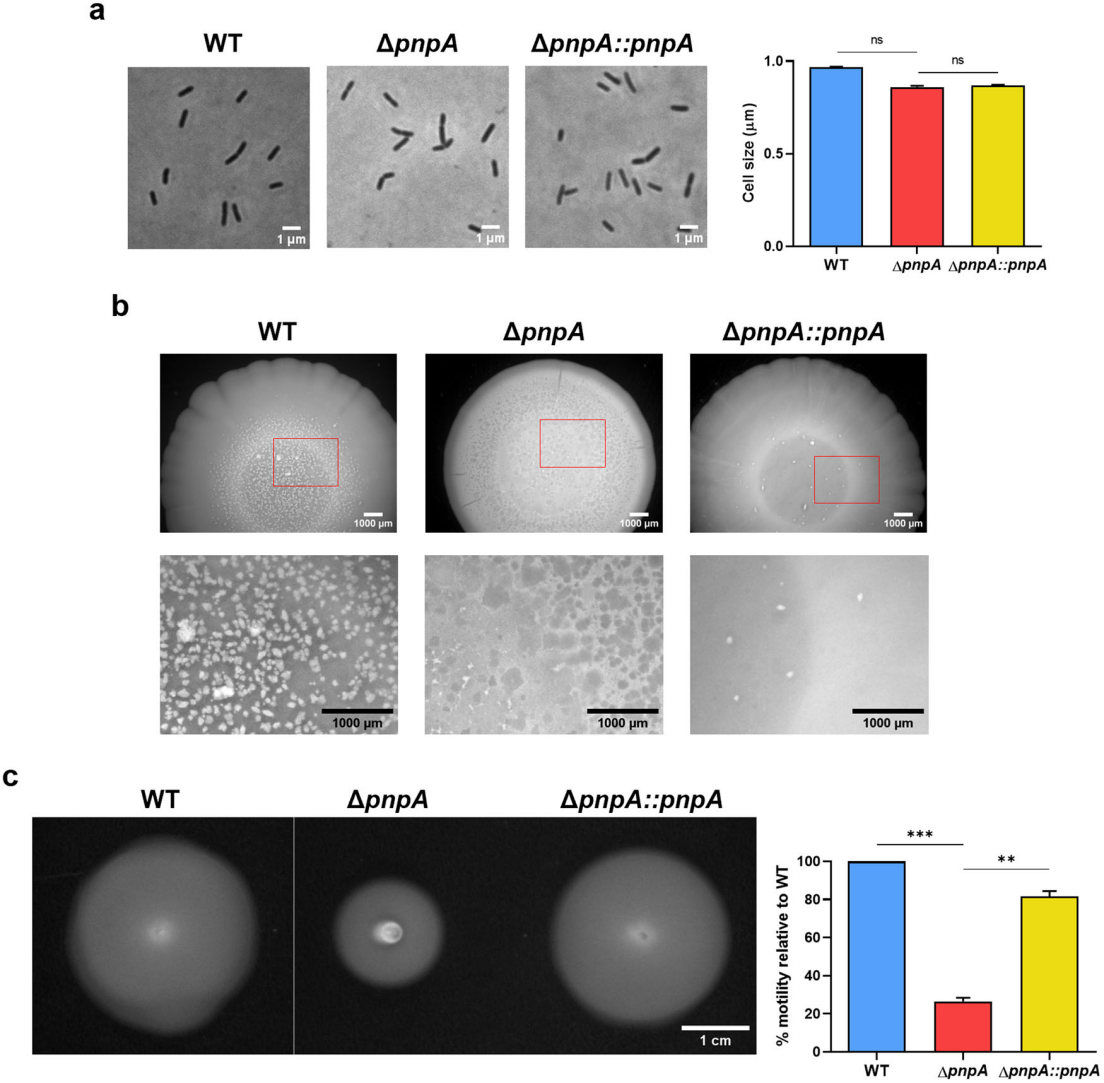
Inactivation of PNPase affects colony morphology and motility of *Listeria monocytogenes*. **a** Representative images of individual bacterial cells at mid-exponential phase (OD_600_ at 0.7–0.8) to assess cell size and morphology. Scale bars, 1 µm (left). Bar-plot of average cell sizes of two independent biological replicates of each strain (right). Data represent mean ± SD. Significance was determined by an unpaired *t* test. ns, not significant; *P < 0.05. **b** Representative images of macrocolony observation using a zoom microscope. Each strain was inoculated on BHI agar plate and incubated at 37 °C for 8 days. The lower panel corresponds to a zoom magnification. Scale bars, 1000 µm. **c** Representative images of swimming motility assessment from bacteria cultures spotted on 0.3% (w/v) BHI agar and incubated at 25 °C for 48 h (left). Bar-plot of swimming areas of each strain (right). Averaged replicate values were transformed as the percentage motility relative to the wild-type. Data represent mean ± SD of three independent experiments. Significance was determined by an unpaired *t* test; **P < 0.01, ***P < 0.005.

Changes in cell size, shape, and motility have been associated with virulence defects of bacterial pathogens as these traits may affect host invasion^41,42^. To determine if the difference in the colonization ability of host cells by the *Listeria monocytogenes* EGD-e (wild-type) and the Δ*pnpA* mutant cultures could result from a phenotypic variation between these strains, we firstly analyzed their microscopic cellular morphology (Fig. 2a). No significant differences in cell size were found between these two strains or the complemented strain. Next, we analyzed the morphology of macrocolonies from all strains grown on BHI agar plates; and striking differences were observed (Fig. 2b). All colonies were characterized by a central core with a concentric ring surrounded by a peripheral area of smooth morphology. Nevertheless, the outer region of the wild-type and complemented macrocolonies presented a lobated edge, while the Δ*pnpA* mutant showed a more regular smooth-growth zone. The most noticeable difference was found in the inner region, as the wild-type showed a granular appearance. Instead, the Δ*pnpA* mutant showed a speckled morphology, which was not confined to the inner core, but it was observed all over the macrocolony and resembled hollow-like structures on the surface (a zoomed image of representative areas of each macrocolony is shown to better visualize the different features) (Fig. 2b). This phenotype was not observed in the complemented strain, which was more similar to the granular appearance found on the wild-type. Since flagella and motility have also been associated to virulence, we decided to evaluate if PNPase could affect the swimming ability of *Listeria*. Cultures of the wild-type and the Δ*pnpA* mutant were spotted on BHI-soft-agar plates (Fig. 2c). Deletion of PNPase resulted in a strain with reduced motility (about 70% less) when compared to the wild-type. This defective phenotype was fully restored in the PNPase-complemented strain.

Collectively, these results showed that the inactivation of PNPase causes phenotypic changes, with differences found in colony morphology and cell motility, which are correlated with the less invasive nature of this strain.

### PNPase affects the formation and structure of *Listeria monocytogenes* biofilms

**Fig. 3:**
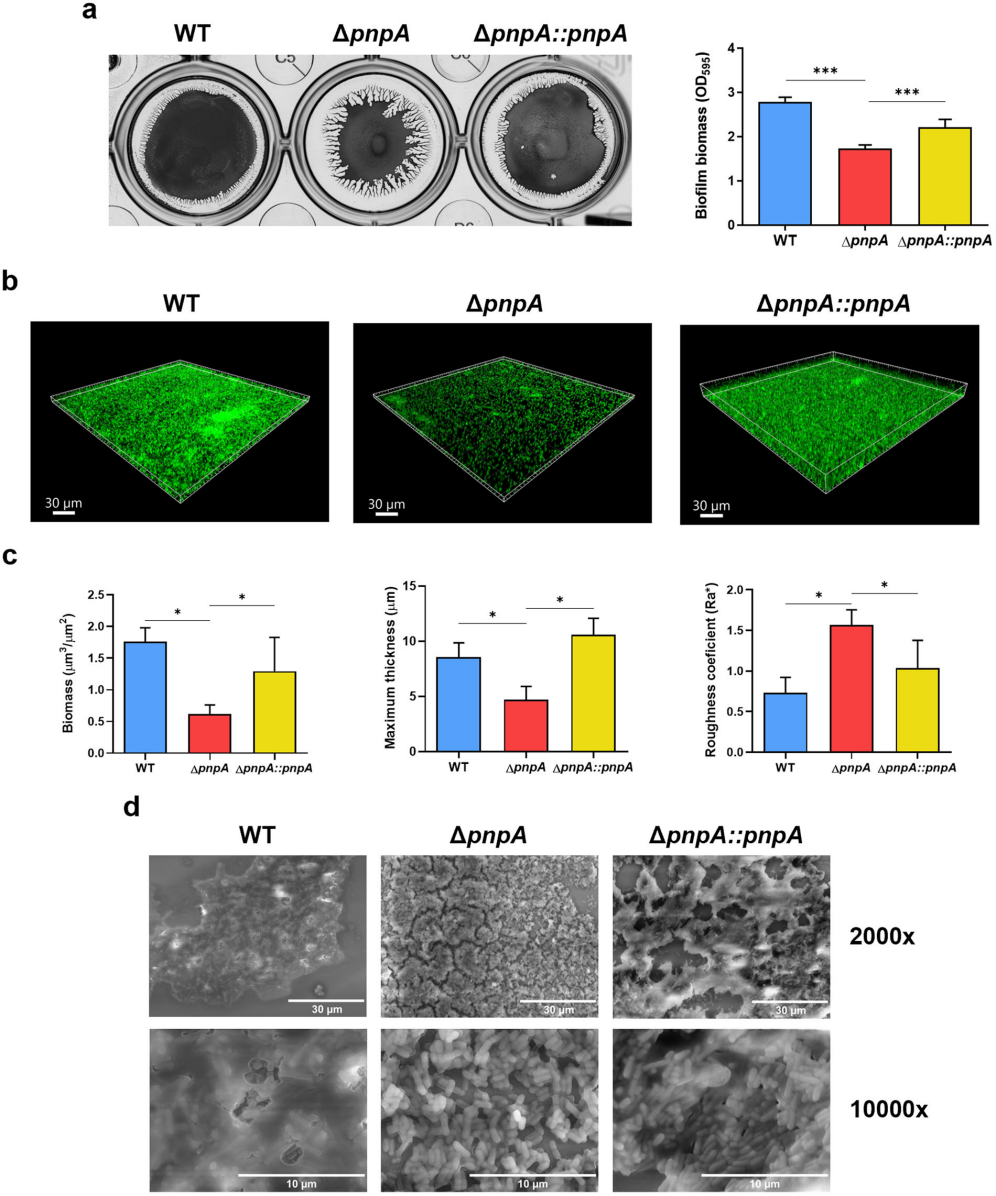
PNPase affects the formation and structure of *Listeria monocytogenes* biofilms. **a** Biofilms were grown statically at 37 °C for 48 h and biofilm biomass was determined using crystal violet staining method. On the left, biofilms were imaged after the addition of crystal violet. On the right, averaged values of the absorbance at 595 nm were plotted. Data represent mean ± SD of three independent experiments. Significance was determined by an unpaired *t* test; ***P < 0.005. **b** Representative three-dimensional structures of the biofilms were reconstructed after acquiring z-stack images by CLSM after growth at 37 °C for 48 h. **c** The biomass, maximum thickness and roughness coefficient of the imaged biofilms were quantified by COMSTAT. Replicate values were averaged and plotted. Data represent mean ± SD of three independent experiments. Significance was determined by an unpaired *t* test; *P < 0.05. **d** Re*p*resentative images of biofilms observed by SEM at 2000x and ×10,000 magnifications.

Colony morphology and motility are two interconnected phenotypes known to affect the formation of bacterial biofilms^43,44^. To determine if the altered morphotype and motility defects found in the PNPase-mutant of *Listeria monocytogenes* could affect biofilm formation, we next compared the amount of biofilm produced by the wild-type, Δ*pnpA* and complemented strains. Bacteria were grown statically in BHI medium at 37 °C in 24-well plates. After the removal of planktonic cells and washing, surface-adhered biofilms were stained with crystal violet (CV). We observed that the *Listeria* PNPase-deletion mutant was clearly defective (40% less) in biofilm formation compared to the wild-type (Fig. 3a). This phenotype was at least partially recovered in the PNPase-complemented strain. Moreover, the morphology of the biofilm observed in the Δ*pnpA* mutant was strikingly different from the biofilms of the two other strains (Fig. 3a, left). Inactivation of PNPase resulted in a biofilm with pronounced streaked-like edges that were not detected in cells expressing PNPase. In addition, we observed that the biofilm in the Δ*pnpA* mutant was more easily detached during the washing steps, suggesting that this biofilm adheres less and is less structured.

To further assess *Listeria* biofilm architecture, we used confocal laser scanning microscopy (CLSM). Biofilms were grown on glass coverslips on 24-well plates for 48 h at 37 °C. Then, the biofilms were gently washed to remove unattached bacterial cells and stained with SYTO™ 9, a cell-permeant fluorescent nucleic acid dye that labels bacteria and extracellular DNA^45^ (eDNA). Z-stacks of each biofilm were obtained and reconstructed into three-dimensional projections. As shown in Fig. 3b, the *Listeria* wild-type strain formed biofilms that resembled a dense lawn of bacteria with some cell aggregates. In contrast, the Δ*pnpA* mutant strain formed a sparse biofilm with many empty areas between individual bacterial cells. In the PNPase-complemented strain, the biofilms resembled the ones formed by the wild-type strain. In addition, the structural differences in biofilm formation were quantified (Fig. 3c). The wild-type biofilm presented more biomass and was significantly thicker than the Δ*pnpA* mutant biofilm. Regarding biofilm roughness, wild-type biofilms presented lower roughness coefficient than Δ*pnpA* mutant biofilm, since wild-type has a more homogeneous aspect, while Δ*pnpA* mutant has reduced biovolume and presents more hollow spaces. Biofilms from the PNPase-complemented strain showed intermediate values of biomass and roughness, although the maximum thickness was higher than the wild-type due to heterogeneity in the z-stack image acquisition. Together, these results validate information obtained from CV assays and together point toward a decrease in Δ*pnpA* mutant biofilm formation.

To better understand the morphological nature of the reduced *Listeria* biofilm formation when PNPase is not expressed, the biofilm architecture of the wild-type, Δ*pnpA* mutant and the PNPase-complemented strain was further studied by Scanning Electron Microscopy (SEM). It was readily observed that biofilms from the wild-type strain exhibited a smooth and hydrated-like surface that was quite different from the rough and cracked surface found in the Δ*pnpA* mutant (Fig. 3d). A higher magnification revealed that wild-type bacteria were mostly covered by the extracellular material, which made it difficult to observe the outlines of individual cells. In sharp contrast, the Δ*pnpA* mutant bacteria were not embedded in a dense extracellular matrix, and cells were easily detected. The PNPase-complemented strain showed a biofilm phenotype more similar to the wild-type with a smooth surface and higher content of extracellular matrix than the Δ*pnpA* mutant. However, we note that complementation was not total as some patches of rough biofilm surface and bacteria not trapped in the extracellular matrix were also detected. The partial complementation of different phenotypes obtained in the Δ*pnpA*::*pnpA* strain is probably related to different levels of PNPase expression in the different assay conditions here tested. This might be a consequence of the cloning strategy used for the amplification of *pnpA* gene for cloning in pPL2 plasmid, that might have missed activator regions or other promoters located in the upstream sequence of *pnpA* (see “Methods”).

Overall, this set of results showed that PNPase affects *L. monocytogenes* biofilms by disturbing the formation of the extracellular matrix. Inactivation of *Listeria* PNPase leads to reduced and thinner biofilms with a significantly lower content of its extracellular matrix.

### PNPase affects the composition of the biofilm matrix

**Fig. 4:**
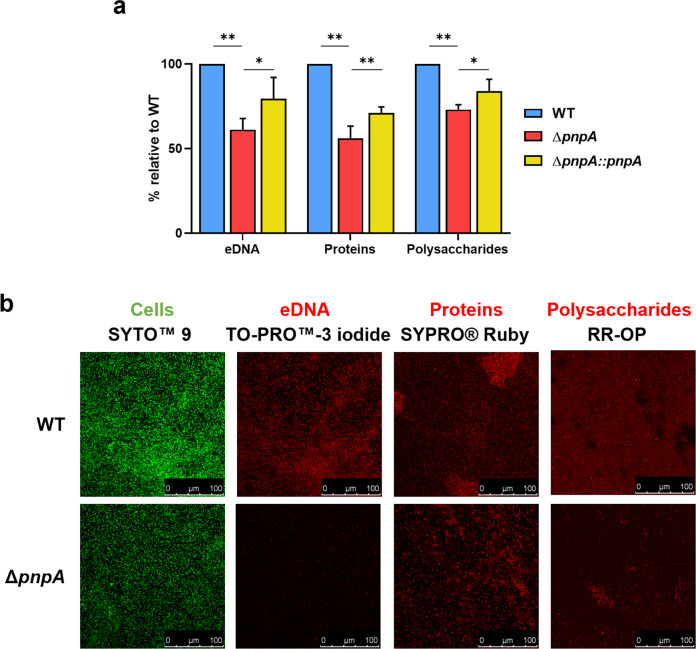
Biofilm extracellular matrix content is reduced in Δ*pnpA* mutant biofilms. **a** Relative quantification of extracellular DNA, protein, and polysaccharide content in the matrix of biofilms grown during 48 h at 37 °C. Averaged replicate values were transformed as the percentage relative to the wild-type. Data represent mean ± SD of three independent experiments. Significance was determined by an unpaired *t* test; *P < 0.05, **P < 0.01. **b** Re*p*resentative images of CLSM analysis of the biofilm matrix composition. SYTO™ 9 was used as a control to dye bacteria, TO-PRO™-3 iodide was used for extracellular DNA staining, SYPRO® Ruby for protein staining, and RR-OP for polysaccharide staining.

The matrix of *Listeria monocytogenes* biofilms is composed of EPS, namely eDNA, proteins and polysaccharides^46^. Following the previous set of results, we investigated the macromolecular biofilm matrix composition of the wild-type, Δ*pnpA,* and complemented strains to understand if the observed differences in biofilm structure and morphology were due to changes in the matrix composition. Biofilms grown for 48 h at 37 °C were scrapped from the abiotic surface and subjected to sonication to separate the EPS from the bacterial cells. After sonication, OD_600_ was measured for further normalization of each matrix component against the total biofilm biomass of each strain. The EPS recovered in the supernatant was further quantified using distinct biochemical techniques according to the component in the study: phenol-chloroform extraction method for eDNA, Bradford’s method for proteins, and phenol-sulfuric acid method for polysaccharides. Remarkably, the results indicate that the amount of the three major components of the *Listeria* extracellular matrix was reduced in the Δ*pnpA* mutant strain when compared to the wild-type (Fig. 4a). Also, we observed that the complemented strain was able to partially recover the wild-type phenotype.

To further validate these results and visualize the different matrix components, 48h-grown biofilms were then analyzed by CLSM after staining with specific dyes for each component, as shown in representative images in Fig. 4b. TO-PRO™-3 iodide fluorescent dye targets nucleic acids but not in bacteria with intact membranes. Thus, it has been used for eDNA detection on the extracellular matrix since it allows the distinction between eDNA and DNA found inside biofilm cells, when used simultaneously with SYTO™ 9 dye^45^. In Fig. 4b, a strong reduction of eDNA levels was detected in Δ*pnpA* mutant biofilm with only few cells being stained, while a stellar-like pattern of eDNA was observed in the wild-type biofilm. FilmTracer™ SYPRO® Ruby Biofilm Matrix dye is used for protein detection^47^ and was able to bind to protein-rich aggregates in the matrix of the wild-type biofilm, but only stained individual cells in the Δ*pnpA* mutant biofilm (Fig. 4b). This demonstrates that there is lower protein content in the extracellular matrix of PNPase-deficient strain. Staining polysaccharides present in the biofilm matrix of *Listeria* was a more challenging procedure. First, we tested the fluorescent dye wheat germ agglutinin (WGA) conjugated with Alexa Fluor 633, commonly used to detect polysaccharides in Gram-positive biofilms, namely in *Staphylococcus aureus*^48^. However, this dye was not effective in the labeling of polysaccharides in *Listeria* biofilms, at least in the conditions here tested. To overcome this difficulty, next we tested ruthenium red (RR), a dye that binds to carbohydrates and was known to stain *Listeria* biofilms^49^. RR is not fluorescent, but when conjugated with non-fluorescent 1,10-phenanthroline (OP) it results in the fluorescent RR-OP complex, previously used to label anionic substrates like chromatin from erythrocytes nuclei^50^. We synthesized RR-OP and tested its ability to label the polysaccharides present in *Listeria* EPS. As observed in Fig. 4b, RR-OP successfully binds to the polysaccharides present in the biofilm matrix. The staining was more intense in the wild-type matrix but stained poorly the Δ*pnpA* mutant, showing that there are lower levels of polysaccharides in the mutant biofilm matrix. To the best of our knowledge, this was the first time RR-OP was used as a fluorescent staining method for polysaccharides on *Listeria* biofilms, providing a new tool that could be useful for the study of bacterial biofilms. Taken together, these results show that inactivation of PNPase leads to the reduction of the EPS in *Listeria* biofilms, affecting the levels of eDNA, proteins and polysaccharides.

### Biofilms of PNPase-depleted strain are more susceptible to antibiotics

**Fig. 5:**
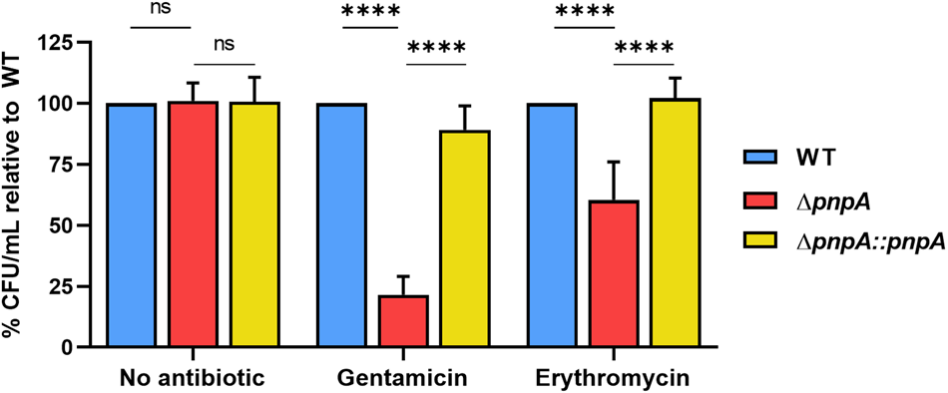
Biofilms of the PNPase-depleted strain are more susceptible to antibiotics. Biofilms grown for 48 h at 37 °C were subjected to a treatment of 24 h with high doses of gentamicin (10X MIC) or erythromycin (100X MIC). As bacterial cultivability control, BHI was added instead of antibiotic. After each treatment, recovered cultivable cells were quantified as CFU/mL. Replicate values were averaged and transformed as the percentage of CFU/mL relative to the wild-type. Data represent mean ± SD of three independent experiments. Significance was determined by two-way ANOVA; ns, not significant; ****P < 0.0001.

Biofilms are more resistant to the action of antimicrobial agents due to their robust structure and to the protective role of the surrounding matrix. Since inactivation of PNPase leads to thinner biofilms with less EPS content, next we evaluated if PNPase-deficient biofilms were less resistant to antibiotics treatment. The two antibiotics used for the treatment of listeriosis were selected, in this case gentamicin and erythromycin (which are considered the first- and second-line therapeutic agents, respectively^51,52^). First, the minimum inhibitory concentrations (MIC) of planktonic cultures grown in BHI broth were determined and revealed no differences between the strains, in terms of bacterial cultivability in BHI-A plates (3 µg/mL for gentamicin and 0.1 µg/mL for erythromycin). Then, wild-type and Δ*pnpA* mutant biofilms grown statically in BHI for 48 h were subjected to a treatment with high doses of each antibiotic. After 24 h, the biofilms were washed, subjected to an ultrasound bath, and scraped to release the attached cells from the abiotic surface. The recovered biofilm cells were plated in BHI agar plates for CFU/mL assessment. In parallel, BHI without antibiotic was used as a control for cellular cultivability. There were no significant differences between wild-type and Δ*pnpA* mutant when no antibiotic was added to the pre-formed biofilms, showing that inactivation of PNPase does not affect cell cultivability in *Listeria* biofilms (Fig. 5). In contrast, each antibiotic treatment resulted in lower cultivability for Δ*pnpA* mutant when compared to the wild-type, although we noted that this reduction was less than 1-log. This effect was more pronounced with gentamicin treatment (Δ*pnpA* strain was 75% less cultivable), although a reduction in cultivability was also observed when erythromycin was used (Δ*pnpA* strain was 40% less cultivable) (Fig. 5). The complemented strain showed nearly full complementation in both antibiotic treatments. These results show that the biofilm of the PNPase mutant is more susceptible to antibiotics than the wild-type. This fully agrees with our previous observations that PNPase-deficient biofilms are less structured and have less extracellular matrix components.

### Transcriptomic analysis of *Listeria* PNPase-deficient biofilms

**Fig. 6:**
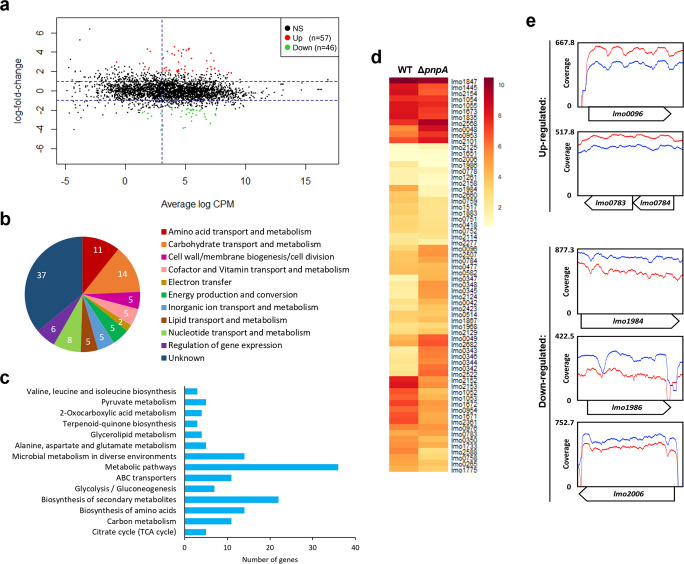
Transcriptomic analysis of PNPase-deficient biofilms. **a** MA scatterplot comparing the expression of transcripts between two biological replicates of *L. monocytogenes* EGD-e wild-type and Δ*pnpA* mutant biofilms. Genes with significantly different expressions are highlighted in red if they are upregulated or in green if they are downregulated in Δ*pnpA* biofilms compared to wild-type. The FDR cut-off is <0.10. The two horizontal lines correspond to the cut-off of a log_2_ fold change of 1, and the vertical line to the cut-off of the log_2_ CPM of 3. NS not significant. **b** Global visualization of differentially expressed genes (DEGs) divided into general biological categories. The number of genes belonging into each category is in white. **c** DEGs with significantly increased enrichment grouped into predicted KEGG pathways. **d** Heatmap of the transcriptional profile of the DEGs. Hierarchical clustering was done to group genes with similar expression pattern in terms of log_2_ RPKM. **e** Read coverage plots of three upregulated (*lmo0096*, *lmo0783*, *lmo0784*) and three downregulated genes (*lmo1984*, *lmo1986*, *lmo2006*). *lmo0783-0784* are shown in operon. Blue line corresponds to wild-type, while red line corresponds to Δ*pnpA* mutant. The *y* axis represents the coverage of reads and the maximum value of each gene is shown. The *x* axis represents the gene position.

To better understand how PNPase affects gene expression in biofilm formation, we compared the transcriptome between Δ*pnpA* mutant and wild-type biofilm cultures. Total RNA was extracted from biofilm cultures grown for 48 h at 37 °C and submitted to RNA-sequencing (RNA-seq). From the RNA-seq data, we assessed the differential transcript expression between Δ*pnpA* mutant and wild-type biofilms by calculating and plotting the fold change for each transcript in a MA scatterplot (Fig. 6a). To determine the differentially expressed genes (DEGs) that were statistically significant we defined the following parameters: a false-discovery rate (FDR) lower than 0.1, a fold change higher than 2, and an expression value of the transcripts (log_2_ CPM) higher than 3. Although the dispersion of the log_2_ fold change values was considerably reduced for most of the transcripts, a total of 103 genes were differentially expressed (Supplementary Table 1), of which 46 were downregulated, and 57 were upregulated (Fig. 6a). Remarkably, when the DEGs were grouped according to their general biological role, we observed that the majority of DEGs was part of either the “Carbohydrate transport and metabolism” (*n* = 14) or the “Amino acid transport and metabolism” (*n* = 11) groups, along with the uncategorized DEGs (*n* = 37) (Fig. 6b). Next, DEGs were mapped to the KEGG database to attribute each gene to its respective pathway, followed by KEGG pathway enrichment analysis (Fig. 6c). Results showed enrichment in pathways involved in “Metabolic pathways” (*n* = 36), “Biosynthesis of secondary metabolites” (*n* = 22) and “Microbial metabolism in diverse environments” (*n* = 14). We can observe a good correlation between the categories ascertain in Fig. 6b and the enriched KEGG pathways described in Fig. 6c: the category “Amino acid transport and metabolism” is supported by enrichment of “Biosynthesis of amino acids”, “Alanine, aspartate and glutamate metabolism”, and “Valine, leucine and isoleucine biosynthesis” pathways; in the same manner, the category “Carbohydrate transport and metabolism” can englobe the KEGG pathways “Carbon metabolism”, “Citrate cycle”, and “Glycolysis/Gluconeogenesis”.

A heatmap was charted, showing the normalized expression values of the DEGs in the wild-type and the Δ*pnpA* mutant strains (Fig. 6d). We then selected six of those differentially expressed transcripts (3 up- and 3 downregulated genes) as examples of our RNA-seq data and plotted their read coverage to assess the sequencing depth in both strains (Fig. 6e). Next, genes that presented higher and lower log_2_ FC, and that have been previously associated with biofilm formation according to the literature were selected as representative genes (Table 1). For instance, inactivation of PNPase led to the upregulation of genes from the quorum sensing system Agr (*lmo0048*, *lmo0049*), genes from the mannose/glucose (*lmo0096*, *lmo0783*, *lmo0784*) and maltose (*lmo2124*, *lmo2125*) transport systems, along with genes involved in the pentose phosphate pathway (*lmo0342*, *lmo0343*, *lmo0345*). The categories that were strongly downregulated by PNPase inactivation were the “Amino acids transport and metabolism” (*lmo1733*, *lmo1835*, *lmo1984*, *lm01986*, *lmo2006*) together with the “Energy production and conversion” (*lmo1052*-*1055*). RNA-seq results were validated by qPCR analysis, using a subset of these representative genes (Table 1). We observed a good correlation between the two techniques, indicating that the qPCR results were consistent with the RNA-seq data.

**Table 1:** Comparison between the values for fold change of selected genes using RNA-seq and qPCR.

	Gene	Product	Biological process	RNA-seq	FDR (corrected *P* value)	qPCR
Upregulated	*lmo0096*	PTS mannose/glucose transporter subunit IIAB, ManL	Carbohydrate transport and metabolism	4.83	8.34 × 10^−3^	2.48
	*lmo0342*	Transketolase, Tkt		14.82	1.82 × 10^−7^	–
	*lmo0343*	Transaldolase, Tal2		19.30	1.62 × 10^−8^	3.46
	*lmo0345*	Ribose 5-phosphate isomerase, Rpi		23.90	9.58 × 10^−9^	3.22
	*lmo0783*	PTS mannose/glucose transporter subunit IIB, MpoB		4.44	1.95 × 10^−2^	–
	*lmo0784*	PTS mannose/glucose transporter subunit IIA, MpoA		4.36	2.13 × 10^−2^	2.71
	*lmo2124*	Maltose/maltodextrin ABC-transport system permease, MalF		10.59	7.78 × 10^−6^	2.00
	*lmo2125*	Maltose/maltodextrin ABC-transporter binding protein, MalE		15.09	2.38 × 10^−7^	3.05
	*lmo0048*	Sensor of histidine kinase, AgrB	Quorum sensing system	8.59	4.36 × 10^−5^	6.73
	*lmo0049*	Autoinducing peptide, AgrD		5.44	4.10 × 10^−3^	–
Downregulated	*lmo1733*	Glutamate synthase subunit beta, GltD	Amino acids transport and metabolism	0.07	2.90 × 10^−7^	0.14
	*lmo1835*	Carbamoyl-P synthetase large subunit, CarB		0.24	1.62 × 10^−2^	–
	*lmo1984*	Acetolactate synthase large subunit, IlvB		0.11	1.30 × 10^−5^	–
	*lmo1986*	Ketol-acid reductoisomerase, IlvC		0.08	1.78 × 10^−6^	0.19
	*lmo2006*	Acetolactate synthase large subunit, AlsS		0.26	2.33 × 10^−2^	–
	*lmo1052*	Pyruvate dehydrogenase subunit E1 alpha, PdhA	Energy production and conversion	0.10	4.94 × 10^−6^	0.56
	*lmo1053*	Pyruvate dehydrogenase subunit E1 beta, PdhB		0.13	8.54 × 10^−5^	0.56
	*lmo1054*	Dihydrolipoamide acetyltransferase E2, PdhC		0.16	5.57 × 10^−4^	–
	*lmo1055*	Dihydrolipoamide dehydrogenase E3, PdhD		0.26	2.40 × 10^−2^	–

Fold changes were calculated as the ratio of Δ*pnpA* mutant to wild-type. Values above 1 correspond to upregulated transcripts while values below 1 correspond to downregulated transcripts. qPCR was performed in triplicate with RNA extracted from, at least, three independent cultures.

Our results have shown that there was an altered expression in biological categories important for biofilm formation in Δ*pnpA* mutant biofilm when compared with the wild-type, and the majority of the DEGs under control of PNPase are integrated in either carbohydrate or amino acids-related processes. These data corroborate our previous set of results showing that PNPase affects the levels of polysaccharides and proteins from the extracellular matrix of *Listeria monocytogenes* biofilms.

### PNPase affects mRNA stability of genes associated to biofilm and virulence

To further assess the role of PNPase in the regulation of the most significant DEGs, we performed rifampicin mRNA stability assays comparing the Δ*pnpA* mutant with wild-type cultures. Due to technical difficulties in performing this technique in biofilms, alternatively we have used stationary phase cultures grown in BHI medium at 37 °C since at this growth stage, bacteria are more physiologically similar to sessile bacteria present in biofilms^53^. The levels of the mRNAs of the genes of interest were evaluated by Northern blot using specific probes.

**Fig. 7:**
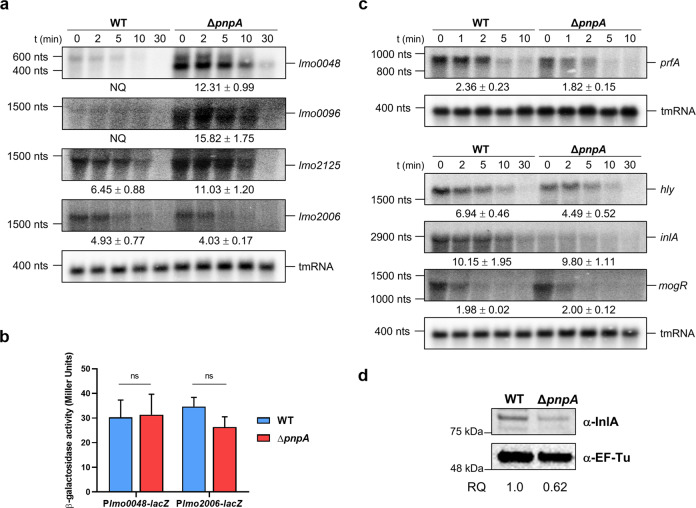
The role of PNPase in the stability and mRNA levels of biofilm and virulence-related genes. **a** Northern blots probed with specific oligos for the detection of *lmo0048*, *lmo0096*, *lmo2125,* and *lmo2006*, comparing RNA extracted from rifampicin-treated cultures of wild-type and Δ*pnpA* mutant strain. RNA stability is shown in minutes under the respective transcript. tmRNA serves as a loading control. A representative gel is shown for each probe from two independent replicates. An RNA size marker is shown on the left of the panel. Two bands were detected with the *lmo0048* probe; half-life quantification shown below the corresponding image is related to the shorter band. Quantification of the upper band showed a stability of 6.2 ± 0.6 in the wild-type and 7.0 ± 0.58 in the Δ*pnpA* mutant. NQ non-quantifiable. **b** β-galactosidase activity of P*lmo0048*-*lacZ* fusion and P*lmo2006*-*lacZ* fusion in wild-type and Δ*pnpA* mutant strain grown in BHI until stationary phase. Data represent mean ± SEM of three independent experiments. Significance was determined by two-way ANOVA; ns, not significant; *P < 0.05. **c** Northern blots probed with specific oligos for the detection of *prfA*, *hly*, *inlA* and *mogR*, comparing RNA extracted from rifampicin-treated cultures of wild-type and Δ*pnpA* mutant strain. RNA stability is shown in minutes under the corresponding image. tmRNA serves as a loading control. A representative gel is shown for each probe from two independent replicates. An RNA size marker is shown on the left of the panel. **d** Western blot analysis of total protein extract using anti-InlA antibody. Anti-EF-Tu antibody was used as loading control. RQ relative quantification.

We observed that inactivation of PNPase resulted in the strong stabilization of mRNAs from upregulated DEGs (Table 1), namely: the quorum sensing-like system *lmo0048/agrB*, the PTS transporter subunit *lmo0096/manL* and the sugar ABC-transporter *lmo2125/malE* (Fig. 7a). This stabilization was particularly obvious when analyzing *lmo0048* and *lmo0096* mRNAs, which showed barely detected levels in the wild-type, not allowing a reliable quantification of the mRNAs half-lives in this strain. We noted that two bands of different sizes were observed with the *lmo0048* probe: a longer band corresponding to the monocistronic mRNA (detected in both the WT and the Δ*pnpA* mutant) and a shorter band corresponding to a decay intermediate (only detected in the Δ*pnpA* mutant). The levels of both mRNA species are higher in the Δ*pnpA* mutant compared to the wild-type strain, being particularly obvious the strong accumulation of the shorter transcript in the absence of PNPase. It is possible that the shorter mRNA might lead to the production of a Lmo0048/AgrB truncated protein, which may still be partially functional and potentially affect biofilm-related pathways. Overall, this set of results demonstrates that PNPase acts as a post-transcriptional regulator by acting as a main enzyme involved in the degradation of mRNAs of upregulated genes identified in our RNA-seq analysis. Contrarily, the mRNA of a selected downregulated gene (*lmo2006*) was observed to decay slightly faster in the Δ*pnpA* mutant, which probably contributes to the observed reduced levels of this transcript in this strain.

In addition, we constructed and analyzed transcriptional fusions to test whether PNPase could affect the transcription of either *lmo0048/agrB* (one upregulated gene) and/or *lmo2006/alsS* (one downregulated gene) (Fig. 7b). Promoter activity of these genes was analyzed using the pTCV plasmid and *lacZ* as a reporter gene^54^. β-galactosidase assays showed that there were no significant differences between wild-type and Δ*pnpA* mutant, what suggests that PNPase is not significantly affecting the transcription of these genes. Considering that we do not observe significative changes in the transcription of *lmo0048* (Fig. 7b), this confirms that the higher levels of the two RNAs in Fig. 7a are due to their stabilization in the absence of PNPase; therefore, PNPase is responsible for their degradation. The shorter transcript of *lmo0048* was not a consequence of increased transcription; instead, this mRNA most likely results from the cleavage of the longer transcript of *lmo0048* by other ribonucleases, being rapidly cleared out by PNPase, which explains why this mRNA was not detected in the wild-type (Fig. 7a).

Since we observed that inactivation of PNPase results in reduced invasion of human cell lines (Fig. 1) and bacterial motility (Fig. 2), we further extended our studies to genes important for these phenotypes, even though they do not appear to be differentially expressed in our biofilm transcriptomic data. Using rifampicin mRNA stability assays and Northern blot analysis, inactivation of PNPase was found to result in lower mRNA levels of the transcriptional activator of virulence genes, *prfA*, along with two *prfA*-regulated genes, namely, *inlA* (codifies for Internalin A) and *hly* (codifies for Listeriolysin O) in stationary phase cultures (Fig. 7c). This regulation appears to be indirectly caused, as the stability of these transcripts was not strongly affected by the absence of PNPase. We also observed reduced protein levels of Internalin A in cell extracts of the Δ*pnpA* mutant, showing that the reduced levels of *inlA* mRNA are correlated with lower levels of protein (Fig. 7d). On the other hand, we observed that PNPase does not affect the levels and stability of *mogR* mRNA, the transcriptional repressor of motility genes (Fig. 7c).

Overall, these results show that PNPase regulates the expression levels of genes involved in biofilm and virulence and supports that PNPase is an important enzyme involved in the post-transcriptional regulation of biofilm formation.

## Discussion

In this work, we present the ribonuclease PNPase as a novel regulator of biofilm formation and virulence in the Gram-positive pathogen *Listeria monocytogenes*. We demonstrate that PNPase is a positive determinant of biofilm production in *L. monocytogenes* EGD-e strain, affecting the biomass, morphology, and structure of the biofilm. The PNPase-deletion strain produced less biofilm biomass, exhibiting a thinner, more rugose and with a dry-looking surface, and less structured biofilm than the wild-type. This was correlated with major defects in the composition of the biofilm extracellular matrix of the Δ*pnpA* mutant, which exhibited a minor content of proteins, polysaccharides, and extracellular DNA, as detected by biochemical assays and microscopy. Indeed, bacteria present in the Δ*pnpA* biofilms are not found to be significantly embedded in a layer of matrix, in sharp contrast to what is observed in the wild-type biofilms where bacteria are surrounded by a thick matrix. These structural defects render a feeble biofilm production in the Δ*pnpA* mutant, which most likely causes the increased susceptibility of *Listeria* biofilms to antibiotics. Moreover, inactivation of *Listeria* PNPase resulted in phenotypic changes in macrocolonies and reduced cell motility, two features widely associated to biofilm formation.

PNPase has been previously associated to biofilm formation in Gram-negative bacteria; however, its exact role is controversial. Whereas PNPase was shown to be a positive regulator of biofilm formation in *E. coli* K-12^38^ and in *Salmonella* Typhimurium^36,37^, another study using *E. coli* C proposed PNPase as an inhibitor of biofilm formation^39^. The effect of PNPase in biofilm production seems thus to be species-dependent, at least in Gram-negative bacteria. Nevertheless, there is a current lack of information about the role of PNPase in the establishment of biofilms in Gram-positive bacteria and our work intended to fill that gap. To better understand how PNPase contributes to the biofilm formation of *L. monocytogenes*, the transcriptomic analysis of the wild-type and Δ*pnpA* mutant biofilms was performed. PNPase was found to affect the expression levels of several genes and multiple regulatory pathways, as was expected from a major post-transcriptional regulator. A total of 103 DEGs affected by PNPase, with 57 and 46 genes showing up- and down-regulation, respectively, were obtained by RNA-seq. The majority of DEGs was part of either the “Carbohydrate transport and metabolism” or the “Amino acid transport and metabolism” groups.

Genes involved in quorum sensing and genes involved in carbohydrate transport and metabolism are amongst the most upregulated genes in *Listeria* biofilms not expressing PNPase (Fig. 6 and Table 1). Initially described in *S. aureus*, the Agr quorum sensing-like system in *L. monocytogenes* is encoded in the *lmo0048-lmo0051* operon^55^. Our results revealed the upregulation of the genes *lmo0048* (encoding a homolog of the *S. aureus* sensor histidine kinase AgrB) and *lmo0049* (encoding a homolog of *S. aureus* autoinducing peptide AgrD) in biofilms of the Δ*pnpA* mutant (Fig. 6 and Table 1). It was previously shown that the Agr operon is important for *Listeria* biofilm formation since Agr mutants, namely Δ*lmo0049* (Δ*agrD*), were affected in adhesion and in the first 24 h of biofilm formation^20,21,56^. The expression of the *L. monocytogenes agr* operon is subjected to temporal regulation, with increasing levels of expression found in the early stages of biofilm growth, and subsequent declining as the biofilm matures^13^. We observe that this temporal regulation of the *agr* system is defective in the absence of PNPase; the high levels of *agrB* and *agrD* transcripts observed in biofilms after 48 h of growth could help explaining the reduction in biofilm biomass found in the Δ*pnpA* mutant.

In addition, several genes involved in sugar transport and bioenergetics were found to be upregulated in the Δ*pnpA* mutant biofilms. These include genes of the phosphoenolpyruvate (PEP)-dependent sugar:phosphotransferase system (PTS) for the high-affinity uptake of both mannose and glucose. The PTS comprises two general phosphotransferase proteins (EI and HPr) and a variable number of sugar-specific enzyme II complexes; EI and HPr transfer phosphoryl groups from PEP to EIIAB that is responsible for phosphorylation of different sugars, with EIICD acting as transporters^57,58^. Our results indicate the upregulation of *lmo0096* (EIIAB^Man/Glu^) from the Man operon and *lmo0783* (EIIA^Man/Glu^) and *lmo0784* (EIIB^Man/Glu^) from the Mpo operon. The upregulation of these PTS may represent an increase in the consumption of glycolytic PEP, thus reducing its availability for the biosynthesis of aromatic amino acids and cell wall precursors^59^. This most probably contributes to the lower biofilm matrix production observed in the *Listeria* Δ*pnpA* biofilms. In agreement with this hypothesis, PTS enzymes are downregulated during biofilm formation in other Gram-positive bacteria, as observed in *Streptococcus pneumoniae*^60^ and in strong and weak biofilm formers of *Enterococcus faecalis*^61^. Further upregulated genes include *lmo2124* and *lmo2125* that encode for the ATP-binding cassette transporter responsible for the uptake of maltose/maltodextrin, which is later used in glycolysis in the form of glucose^62^. We also observed the upregulation of seven genes from the *gol* operon (*lmo0341-0351*), which encode for enzymes involved in the non-oxidative phase of the pentose phosphate pathway (*lmo0342*, *lmo0343*, and *lmo0345*), in glycerol metabolism (*lmo0344*, *lmo0347*, and *lmo0348*) and in glycolysis (*lmo0346*)^63,64^. Altogether, the higher expression of these genes suggests that the metabolism of carbohydrates is focused preferentially on energy production rather than on biosynthetic pathways. This could further help to explain the low biosynthesis of matrix components and consequent defective biofilm formation observed in the absence of PNPase.

Among the downregulated genes found in PNPase-defective biofilms, it was similarly possible to identify metabolic pathways affecting biofilm formation (Fig. 6 and Table 1). A major category was amino acid metabolism, and included the genes *lmo1984* (*ilvB*), *lmo1986* (*ilvC*), *lmo2006* (*alsS*), which are involved in the synthesis of branched-chain amino acids such as isoleucine, leucine, and valine. These amino acids have been shown to promote robust biofilm formation in different bacteria: for example, high levels of leucine and valine were found in *E. coli* biofilms^65,66^; they are among the biofilm-promoting amino acids identified in *Pseudomonas aeruginosa*^67^; and, the metabolism of isoleucine, leucine, and valine is essential during *Bifidobacterium bifidum* biofilm formation^68^. Another downregulated gene involved in amino acid metabolism is *lmo1733* (*gltD*), which encodes the smaller subunit of the glutamate synthase and is responsible for the conversion of glutamine into glutamate. This amino acid was also found to affect biofilm formation, with defects in this metabolic pathway leading to reduced adhesion to solid surfaces and, consequently, less biofilm formation in *L. monocytogenes*^69^ and *Bacillus subtilis*^70^. Overall, the reduced expression of these amino acid genes agrees with the lower protein content of the extracellular matrix and less structured biofilm observed in the Δ*pnpA* mutant. In addition, another downregulated set of genes consists of the *lmo1052-1055* operon encoding the pyruvate dehydrogenase complex (*pdh* operon), responsible for the conversion of pyruvate to acetyl-CoA. In *Streptococcus suis*, protein expression of PDH was higher in the biofilm state than in planktonic^71^ and biofilm formation was significantly decreased after PDH deletion^72^.

Rifampicin mRNA stability assays and Northern blot analysis revealed that PNPase is involved in the degradation of at least a representative subset of the upregulated genes identified in the transcriptomic analysis of biofilms. In the Δ*pnpA* mutant strain, a strong stabilization in the transcripts from *lmo0048/agrB*, *lmo0096/manL* and *lmo2125/malE* was observed when compared to the wild-type strain. The high levels of these transcripts do not seem to be consequence of increased transcription in the absence of PNPase as a transcriptional fusion of promoter from *lmo0048* with *lacZ* showed no significant differences in β-galactosidase activity between the wild-type and the Δ*pnpA* mutant strain. These results confirm PNPase as an important post-transcriptional regulator of genes involved in biofilm formation, supporting that the upregulated DEGs are a consequence of the increased stability of these transcripts in the absence of PNPase. The higher levels of these mRNAs may potentiate translation of the corresponding proteins, therefore affecting biofilm formation pathways.

On the other hand, it was observed that the mRNA of *lmo2006/alsS* decayed slightly faster in the Δ*pnpA* mutant. This suggests that PNPase may act indirectly to control the stability of mRNAs from downregulated DEGs. For instance, PNPase could be controlling the levels of a repressor of these transcripts (like sRNAs)^73,74^, or PNPase may be protecting these mRNAs from degradation by other ribonucleases, similarly to what was described for RNase II in *E. coli*^75^.

Furthermore, PNPase was found to affect the expression of genes important for *Listeria* virulence. Strikingly, the Δ*pnpA* mutant showed reduced mRNA levels of the transcriptional activator of virulence genes, *prfA*, which could imply lower levels of PrfA protein. Since PrfA is autoregulated, it seems likely that lower levels of PrfA could lead to a reduction in the expression of virulence factors^76^. As a matter of fact, we observed that *inlA*/Internalin A and *hly*/Listeriolysin O virulence factors presented reduced values of mRNA in the Δ*pnpA* mutant without major differences in the mRNA half-lives between the Δ*pnpA* mutant and the wild-type strain. Importantly, we found decreased levels of Internalin A in the absence of PNPase (Fig. 7). The lower expression of these virulence factors could indeed help explaining the reduced invasion of human cell lines by the Δ*pnpA* mutant. Moreover, the decreased expression of PrfA likely contributes for the reduced levels of biofilms observed in the Δ*pnpA* mutants. Previous reports highlighted that PrfA and members of the PrfA-regulon act as positive determinants of biofilm production in *Listeria monocytogenes*, given that mutants were defective in surface-adhered biofilm formation^23,77,78^. It was proposed^23^ that these defects may result from alterations in cell surface that are detrimental for biofilm development, as PrfA regulates the expression of cell surface factors (like Internalin A) and secreted proteins (like Listeriolysin O), whose mRNA levels were also reduced in the Δ*pnpA* mutant. Finally, we also tested if PNPase was involved in the regulation of MogR, the transcriptional repressor of flagellar motility genes. However, PNPase was not found to affect *mogR* mRNA levels, which means the defect in motility observed in Δ*pnpA* mutant strain seems to be independent of MogR. Therefore, PNPase-dependent regulation of bacterial motility is more complex than anticipated.

*L. monocytogenes* is a major foodborne pathogen and the causative agent of listeriosis, a high fatality rate bacterial infection in immunocompromised patients^9^. Contamination of food products due to the presence of biofilms in food-processing environments causes an economic burden worldwide^79^. We identified the RNA-binding protein and ribonuclease PNPase as an important regulator of *L. monocytogenes* pathogenicity, affecting not only invasion of host’s cells but also its biofilm formation. Furthermore, we uncovered PNPase-dependent regulatory pathways important for the development of *Listeria* biofilms, highlighting the importance of post-transcriptional regulation for adaptation to biofilm lifestyle of pathogenic bacteria. Other RNA-binding proteins, such as Hfq and CsrA, have been shown to be involved in the control of biofilm formation, namely through their effect on motility, extracellular polysaccharide production, c-di-GMP production, among others^29,80–82^.

In conclusion, we have demonstrated for the first time that PNPase is a post-transcriptional regulator of *Listeria monocytogenes* biofilm formation, with inactivation of PNPase leading to reduced biofilm production. Our results show that PNPase affects multiple metabolic pathways, from carbohydrates to amino acids metabolism and quorum sensing system, affecting the production of extracellular matrix and consequently the formation of *Listeria* biofilms on abiotic surfaces. This work highlights the importance of RNA-binding proteins in the adaptation to a sessile lifestyle and expands our knowledge of biofilm formation in Gram-positive pathogenic bacteria.

## Methods

### Bacterial strains and growth conditions

All bacterial strains and plasmids are listed in Supplementary Table 2. Strains used in this study are *Listeria monocytogenes* EGD-e strain (wild-type) and its isogenic mutant Δ*pnpA* (carrying a null mutant of PNPase/Lmo1331). PNPase complementation was obtained by cloning of a PCR fragment that contains the total sequence of *pnpA* ORF with 343 bps upstream and 162 bps downstream (primers pPL2-pnpA-BamHI and pPL2-pnpA-SalI) into the pPL2 vector following integration into the Δ*pnpA* mutant strain, resulting in the complemented strain Δ*pnpA::pnpA*^35^. Strains were routinely grown in Brain Heart Infusion (BHI) medium (BD Difco™). For Δ*pnpA::pnpA* growth, chloramphenicol was added to a final concentration of 10 μg/mL. Kanamycin was included at 50 μg/mL when either *E. coli* or *L. monocytogenes* strains carried pTCV derivatives.

### Infection assays

Two human cell lines were used: HeLa (ATCC® CCL-2™) and HepG2 (ATCC® HB-8065™). Cells were routinely grown to 80-90% confluency in Dulbecco’s modified Eagle’s medium (DMEM) (Biowest) with high glucose and l-glutamine, supplemented with 10% fetal bovine serum (FBS) (Biowest). HeLa (1 × 10^5^ cells/well) and HepG2 (1 × 10^5^ cells/well) were seeded in 24-well culture plates and incubated at 37 °C, in a 5% CO_2_ atmosphere for 24 h before infection (48 h for HepG2). Internalization assay protocol was adapted from ref. ^40^. Briefly, bacterial suspensions were diluted to the indicated multiplicity of infection (MOI), added to the cells and incubated for 1 h at 37 °C. After that, DMEM with gentamicin (40 μg/mL) was added and incubated for 1 h at 37 °C. After washing, human cells were lysed with 0.1% (v/v) Triton X-100, and serial dilutions were performed to determine the number of recovered intracellular bacteria, expressed as colony-forming unit per mL (CFU/mL).

### Phase contrast microscopy

Images were acquired on a Leica DM 6000B upright microscope equipped with an Andor iXon 885 EMCCD camera and controlled with the MetaMorph V5.8 software (Molecular Devices LLC), using the 100×1.4 NA oil immersion objective plus a 1.6× optivar, and the Phase Contrast optics. Image processing was performed using Fiji software. Uncropped images are provided in Supplementary Fig. 1.

### Colony morphology and motility assay

Images from the macrocolonies were acquired on a Zeiss Axio Zoom.V16 stereo microscope equipped with a Zeiss Axiocam 503 mono CCD camera and controlled with the Zeiss Zen 2.1 (blue edition) software (Zeiss), using the 1 × 0.25 NA objective and the Bright Field optics. Image processing was performed using Fiji software. For determination of swimming motility, a bacterial suspension at OD_600_ ∼ 0.7 was inoculated on BHI-soft-agar plates (0.3% (w/v) agar). Plates were incubated upwards at 25 °C for 48 h and were photographed using Epi-white feature on Gel Doc XR (Bio-Rad) (Supplementary Fig. 2).

### Biofilm formation assay

Overnight grown cultures were washed once in fresh BHI, diluted 1:100 and 500 µL were added to each well of a 24-well plate. Plates were incubated for 48 h at 37 °C, in static conditions. After incubation, biofilms were washed with Dulbecco’s Phosphate Buffered Saline 1× w/o Ca^2+^/Mg^2+^ (DPBS) (Biowest) and dried at 37 °C, followed by the addition of 0.1% (w/v) crystal violet solution. Plate was washed and after being completely dry, it was scanned using Image Scanner III (GE). To quantify the biomass of each biofilm, 33% (v/v) acetic acid was added to solubilize the CV and A_595_ was measured using a spectrophotometer (BioPhotometer plus, Eppendorf).

### Scanning electron microscopy

Sterile round coverslips were placed in each well of the 24-well plate and the bacterial suspensions were prepared as described in “Biofilm formation assay”. Following a 48-h incubation at 37 °C, biofilms were washed with DPBS. Then, they were fixed with the fixation solution (2.5% (v/v) glutaraldehyde, 1% formaldehyde and 0.1 M cacodylate buffer, pH 7.4) and washed with 0.1 M cacodylate buffer. The samples were progressively dehydrated using increasing concentrations of ethanol (50%, 70%, 90%, 100%), and finally tert-butyl alcohol was added for 2 h, followed by freezing at −20 °C until posterior use. The samples were lyophilized under vacuum conditions and kept at room temperature until the SEM analyses were performed. Samples were coated with gold (∼6 nm thickness) using an electron sputter (Cressington 108) during 15 s at 10 mA and imaged in a Hitachi SU8010 scanning electron microscope, at 1.5 kV for a WD of 6 mm.

### Confocal laser scanning microscopy

Biofilms were formed for 48 h on sterile glass round coverslips and then washed with DPBS. To observe the biofilm thickness, biofilms were stained with 3 µM of SYTO™ 9 fluorescent dye (Thermo Fisher Scientific) and images were acquired by CLSM using the 488 nm Ar^+^ laser line (emission collected at 500–590 nm) and scanning z-stacks at a scanning step size of 1.5 µm. To observe the biofilm matrix extracellular DNA and bacterial cells, SYTO™ 9 (Thermo Fisher Scientific) at 3 µM in DPBS was added first, followed by TO-PRO™-3 iodide (Thermo Fisher Scientific) fluorescent dye at 4 µM in DPBS^45^. Images were acquired using the 488 nm Ar^+^ laser line (emission collected at 500–590 nm) and the 633 nm He–Ne laser line (emission collected at 645–795 nm), respectively. FilmTracer™ SYPRO® Ruby Biofilm Matrix stain (Thermo Fisher Scientific) was used for protein staining^47^. Images were acquired using the 476 nm Ar^+^ laser line (emission collected at 600–740 nm). Ruthenium red-Phenanthroline (RR-OP) complex was used to stain polysaccharides in the biofilm structure. The synthesis of RR-OP complex followed a modified protocol by Bertolesi et al.^50^. Briefly, 20 mg of ruthenium red (RR) was dissolved in 10 mL of distilled water, and then 10 mg of 1,10-phenanthroline (OP) was added to the solution. Next, the mixture was heated at 100 °C for 50 min. The complex was obtained as a dark green solution, which was further used to stain biofilms at 2 mg/mL. Images were acquired using the 458 nm Ar^+^ laser line (emission collected at 490–625 nm). In all cases, a Leica TCS SP5 inverted microscope with a 63× water (1.2 numerical aperture) apochromatic objective was used. Images were collected with 512 × 512 pixels at a scan rate of 100 Hz, except for the z-stack measurements that were performed at 200 Hz. Three-dimensional images of the biofilms were constructed using Imaris software (Bitplane). Biofilm biomass, maximum thickness and roughness coefficient were quantified from the z-stacks using COMSTAT software^83^.

### Quantification of matrix components

An adaptation of the quantification method described previously by Combrouse et al.^84^ was followed for the extraction and quantification of biofilm matrix components. After collecting the biofilms, each suspension was sonicated on ice using a probe sonicator (UP 200 s, Dr. Hielscher GmbH). After sonication, OD_600_ was measured for further normalization and suspensions were centrifuged at 3100 × *g* to collect the supernatants for quantification. The concentration of polysaccharides in the biofilm matrix was determined by phenol-sulfuric acid method, using glucose as standard^85,86^. The protein concentrations were quantified using the Bradford reagent^87^ (Bio-Rad), with bovine serum albumin as standard. The extracellular DNA of the biofilm matrix was obtained by phenol-chloroform extraction method^88^ and the concentration was determined using NanoDropOne^c^ (Thermo Fisher Scientific).

### Eradication of biofilms using antibiotics

Minimum inhibitory concentrations (MIC) of gentamicin and erythromycin in wild-type and Δ*pnpA* mutant strains were determined using an adaptation of the twofold broth microdilution method in microtiter plates with BHI at 37 °C. MIC was determined by the lowest concentration of antibiotic-inhibiting bacterial growth. For the eradication assays, after washing, the antibiotic solutions (in BHI) were added to 48 h-old biofilms and incubated for 24 h at 37 °C. Then, each well was washed with sterile DPBS, followed by sonication in an ultrasound bath for 5 min. Biofilm was removed and recovered and serial dilutions were done in sterile DPBS and plated in BHI agar plates. CFU/mL was assessed after overnight incubation at 37 °C.

### RNA extraction of biofilms

Biofilms were washed with DPBS, followed by the addition of Buffer A (10% (v/v) glucose, 12.5 mM Tris pH 7.5, 10 mM EDTA in H_2_O) to each well and the biofilm was collected. Cell lysis was performed on FastPrep®-24 (MP Bio) followed by phenol-chloroform method and precipitation with ethanol^35^. Turbo DNase (Thermo Fisher Scientific) was used to remove genomic DNA. RNA quality and integrity were analyzed by agarose gel electrophoresis and on Qubit™ 4 Fluorometer (Thermo Fisher Scientific).

### RNA-sequencing and data analysis

Total RNA samples of two biological replicates from wild-type and Δ*pnpA* mutant strains were sequenced at STAB VIDA (Portugal) with an Illumina HiSeq 4000 platform (paired-end, 150 bp read length, 20 M reads). The cDNA library construction was carried out using Kapa RNA Hyper Prep Library preparation kit with QIA FastSelect -5S/16 S/23 S rRNA depletion. RNA-seq data was analyzed following the workflow described in Pobre and Arraiano^89^. Reads were mapped against *L. monocytogenes* genome (NC_003210.1 downloaded from NCBI genome database) using Bowtie2 program. Visualization of the data was performed with Artemis Genome Browser. The differential expression analysis was done with the R package edgeR. We considered all transcripts with a False Discovery Rate (FDR) correction of the *P* value lower than 0.1 as significant and we further filtered our results using the expression values (log_2_ CPM) higher than 3 and a fold change between two samples higher than 2. Due to the small number of biological replicates for each strain, we opted for the use of a moderate FDR correction of the *P* value lower than 0.1 instead of a more stringent value of 0.05 to avoid underestimation of DEGs. The functional annotation was performed with DAVID functional annotation tool.

### Quantitative real-time PCR

cDNA was synthesized from 1 µg of purified RNA using the SensiFAST™ cDNA Synthesis Kit (Bioline). Reverse transcription coupled to a quantitative PCR (qPCR) was performed with a Real Time Thermal Cycler qTower system (Analytik Jena) and using SensiFAST SYBR No-ROX kit (Bioline) according to the supplier’s instructions. Primers for qPCR are listed in Supplementary Table 3. Relative quantification of gene expression was calculated with the 2^-ΔΔCt^ method and using *gyrA* (*lmo0007*) as the housekeeping reference gene.

### RNA stability assay and northern blot analysis

To determine RNA stabilities, bacterial cultures were grown to stationary phase (OD_600_ ∼ 3) in BHI medium at 37 °C, with agitation. Transcription was blocked by adding rifampicin to a final concentration of 500 µg/mL. Timepoint zero was collected right before the addition of rifampicin. Culture samples were collected at defined timepoints and mixed with 0.2 volumes of RNA stop buffer (1:20 acidic phenol:ethanol solution), followed by centrifugation and flash-freeze. After resuspending pellets in Buffer A, cell lysis was performed on FastPrep®-24 (MP Bio), followed by phenol-chloroform extraction and precipitation with ethanol. For Northern blot analysis, 10–40 µg of total RNA was fractionated in 1% agarose formaldehyde-denaturing gel in MOPS buffer. RNAs were transferred onto Hybond-N^+^ membrane (Cytiva) and UV crosslinked by UV irradiation using a UVC 500 apparatus (Amersham Biosciences). DNA oligonucleotide probes were labeled with [γ-^32^P]-ATP (PerkinElmer) at the 5’ end using T4 polynucleotide kinase (Thermo Fisher Scientific). Radiolabelled probes were purified on G25 Microspin columns (Cytiva). Membranes were hybridized in PerfectHyb Plus Hybridization Buffer (Sigma-Aldrich) at 42 °C, overnight, and analyzed using FUJI TLA-5100 scanner (Fujifilm). The half-lives of RNA were determined by linear regression using the logarithmic of the percentage of RNA remaining versus time, considering the amount of RNA at 0 min as 100%. Unprocessed images of the Northern blots are provided in Supplementary Figs. 3 and 4. Oligonucleotide probes used in this work are listed in Supplementary Table 3.

### Transcriptional fusions

To construct the P*lmo0048*-*lacZ* and P*lmo2006*-*lacZ* transcriptional fusion vectors, the promoter region of each gene was amplified using PCR with the appropriate primer pairs (Supplementary Table 3). The PCR product was digested with *EcoRI* and *BamHI* (Thermo Fisher Scientific) and inserted into the corresponding site of the low copy-number pTCV-based expression vector carrying promoter-less *E. coli lacZ* (pTCV-*lacZ*)^54^. The resulting plasmid constructs were transformed into *E. coli* S17-1, which was used for conjugation^90^ with *L. monocytogenes* EGD-e WT and the isogenic Δ*pnpA* mutant.

### β-Galactosidase assay

Promoter activity was analyzed by measuring the β-galactosidase activity using the pTCV plasmid^54^. Cells were grown to stationary phase (OD_600_ ∼ 3) in BHI medium at 37 °C, with agitation. Collected samples (1 mL) were centrifuged and pellets were flash-frozen. Pellets were resuspended in 1 mL of Z Buffer (60 mM Na_2_HPO_4_, 40 mM NaH_2_PO_4_, 10 mM KCl, 1 mM MgSO_4_, 50 mM β-mercaptoethanol, pH 7.0) and OD_600_ was measured. Cells were permeabilized with 0.5% toluene and 4.5% ethanol for 5 min at 30 °C in a water bath. To determine the β-galactosidase activity, 200 µL of Z buffer containing 4 mg/mL o-nitrophenyl-β-d-galactopyranoside (ONPG, Sigma-Aldrich) was added to each sample, followed by incubation at 30 °C in a water bath. Reactions were stopped by adding 500 µL 1 M NaCO_3_, and the time was recorded. Absorbance at 420 nm was measured after centrifugation for 5 min at 21,000 × *g*. The β-galactosidase activity (Miller units) was calculated as (1000 × A_420_)/(T × V × OD_600_). T, reaction time (in minutes); V, volume of bacteria (in mL).

### Total protein extraction

For total protein extraction, bacterial cultures were grown to stationary phase (OD_600_ ∼ 3) in BHI medium at 37 °C, with agitation, followed by centrifugation. Cell lysis was performed on FastPrep®-24 (MP Bio) and protein extract was recovered from the supernatant. Protein quantification was performed using Bradford’s reagent, as above.

### Immunoblotting

Protein samples were loaded into a Bolt™ 4–12% Bis-Tris Plus Gel (Invitrogen™) with MOPS 1X buffer supplemented with Bolt™ Antioxidant (0.25%), and a Mini Gel Tank system (Invitrogen™) was used. Transference to nitrocellulose membranes was performed using the Mini Blot Module (Invitrogen™). For protein detection, membranes were blocked for 1 h with blocking solution (TBS + 0.1% Tween-20 (TBS-T) + 5% of non-fat powdered milk) and incubated overnight at 4 °C with the following primary antibodies: rabbit α-InlA 1:5000 (Cusabio) or rabbit α-EF-Tu 1:40000 (Abcam) in TBS-T. The secondary antibody goat α-rabbit IgG-HRP 1:20,000 (Sigma) was incubated for 1 h at 4 °C. Detection was performed by chemiluminescence using Western Lightning® Plus-ECL Enhanced Chemiluminescence Substrate (PerkinElmer) in iBright™ CL1500 Imaging System (Supplementary Fig. 5). Images were quantified using Fiji software.

### Statistical analysis

Experimental data were analyzed using GraphPad Prism version 8.0.1 (GraphPad Software). The Student’s *t* test was used to determine the statistical significance for most experiments. A comparison between antibiotic treatment and control was performed using two-way ANOVA. Data are presented as the mean ± standard deviation (SD) or as the mean ± standard error of the mean (SEM). A *P* value of <0.05 was considered statistically significant.

### Reporting summary

Further information on research design is available in the Nature Research Reporting Summary linked to this article.

## Supplementary information

Supplemental Information

Reporting Summary

supplementary table 1

## Data Availability

The data discussed in this publication have been deposited in NCBI’s Gene Expression Omnibus and are accessible through GEO Series accession number GSE210097.

## References

[1] da Silva RAG, Afonina I, Kline KA (2021). Eradicating biofilm infections: an update on current and prospective approaches. Curr. Opin. Microbiol..

[2] Penesyan A, Paulsen IT, Kjelleberg S, Gillings MR (2021). Three faces of biofilms: a microbial lifestyle, a nascent multicellular organism, and an incubator for diversity. NPJ Biofilms Microbiomes.

[3] Vestby LK, Grønseth T, Simm R, Nesse LL (2020). Bacterial biofilm and its role in the pathogenesis of disease. Antibiotics.

[4] Shi X, Zhu X (2009). Biofilm formation and food safety in food industries. Trends Food Sci. Technol..

[5] Sauer K (2022). The biofilm life cycle: expanding the conceptual model of biofilm formation. Nat. Rev. Microbiol..

[6] Flemming HC, Wingender J (2010). The biofilm matrix. Nat. Rev. Microbiol..

[7] Jefferson KK (2004). What drives bacteria to produce a biofilm?. FEMS Microbiol. Lett..

[8] Hall CW, Mah TF (2017). Molecular mechanisms of biofilm-based antibiotic resistance and tolerance in pathogenic bacteria. FEMS Microbiol. Rev..

[9] European Food Safety Authority & European Centre for Disease Prevention and Control. The European Union One Health 2019 Zoonoses Report. *EFSA J*. **19**, 6406 (2021). 10.2903/j.efsa.2021.6406 PMC7913300 33680134

[10] Radoshevich L, Cossart P (2018). *Listeria monocytogenes*: towards a complete picture of its physiology and pathogenesis. Nat. Rev. Microbiol..

[11] Gandhi M, Chikindas ML (2007). *Listeria*: a foodborne pathogen that knows how to survive. Int. J. Food Microbiol..

[12] Ferreira V, Wiedmann M, Teixeira P, Stasiewicz MJ (2014). *Listeria monocytogenes* persistence in food-associated environments: epidemiology, strain characteristics, and implications for public health. J. Food Prot..

[13] Gray J (2021). Colonisation dynamics of *Listeria monocytogenes* strains isolated from food production environments. Sci. Rep..

[14] Lee BH (2019). Biofilm formation of *Listeria monocytogenes* strains under food processing environments and pan-genome-wide association study. Front. Microbiol..

[15] Bai X (2021). Biofilm-isolated *Listeria monocytogenes* exhibits reduced systemic dissemination at the early (12–24 h) stage of infection in a mouse model. NPJ Biofilms Microbiomes.

[16] Chae MS, Schraft H (2000). Comparative evaluation of adhesion and biofilm formation of different *Listeria monocytogenes* strains. Int. J. Food Microbiol..

[17] Guilbaud M, Piveteau P, Desvaux M, Brisse S, Briandet R (2015). Exploring the diversity of *Listeria monocytogenes* biofilm architecture by high-throughput confocal laser scanning microscopy and the predominance of the honeycomb-like morphotype. Appl. Environ. Microbiol..

[18] Rieu A (2008). *Listeria monocytogenes* EGD-e biofilms: no mushrooms but a network of knitted chains. Appl. Environ. Microbiol..

[19] Lemon KP, Higgins DE, Kolter R (2007). Flagellar motility is critical for *Listeria monocytogenes* biofilm formation. J. Bacteriol..

[20] Rieu A, Weidmann S, Garmyn D, Piveteau P, Guzzo J (2007). *agr* system of *Listeria monocytogenes* EGD-e: role in adherence and differential expression pattern. Appl. Environ. Microbiol..

[21] Zetzmann M (2019). Characterization of the biofilm phenotype of a *Listeria monocytogenes* mutant deficient in *agr* peptide sensing. Microbiologyopen.

[22] Hsu CY (2020). Genomic differences between *Listeria monocytogenes* EGDe isolates reveal crucial roles for SigB and wall rhamnosylation in biofilm formation. J. Bacteriol..

[23] Lemon KP, Freitag NE, Kolter R (2010). The virulence regulator PrfA promotes biofilm formation by *Listeria monocytogenes*. J. Bacteriol..

[24] Travier L (2013). ActA promotes *Listeria monocytogenes* aggregation, intestinal colonization and carriage. PLoS Pathog..

[25] Van Der Veen S, Abee T (2010). Importance of SigB for *Listeria monocytogenes* static and continuous-flow biofilm formation and disinfectant resistance. Appl. Environ. Microbiol..

[26] Dramsi S (1995). Entry of *Listeria monocytogenes* into hepatocytes requires expression of inlb, a surface protein of the internalin multigene family. Mol. Microbiol..

[27] Mika F, Hengge R (2013). Small regulatory RNAs in the control of motility and biofilm formation in *E. coli* and *Salmonella*. Int. J. Mol. Sci..

[28] Parker A, Cureoglu S, De Lay N, Majdalani N, Gottesman S (2017). Alternative pathways for *Escherichia coli* biofilm formation revealed by sRNA overproduction. Mol. Microbiol..

[29] Condinho, M. et al. The role of RNA regulators, quorum sensing and c‐di‐GMP in bacterial biofilm formation. *FEBS Open Bio.* 1–17 10.1002/2211-5463.13389 (2022). 10.1002/2211-5463.13389 PMC10240345 35234364

[30] Lim B, Beyhan S, Meir J, Yildiz FH (2006). Cyclic-diGMP signal transduction systems in *Vibrio cholerae*: modulation of rugosity and biofilm formation. Mol. Microbiol..

[31] dos Santos RF (2018). Major 3′–5′ exoribonucleases in the metabolism of coding and non-coding RNA. Prog. Mol. Biol. Transl. Sci..

[32] Hu J, Zhu M-JJ (2015). Defects in polynucleotide phosphorylase impairs virulence in *Escherichia coli* O157:H7. Front. Microbiol..

[33] Chen R (2016). Polynucleotide phosphorylase regulates multiple virulence factors and the stabilities of small RNAs RsmY/Z in *Pseudomonas aeruginosa*. Front. Microbiol..

[34] Rosenzweig JA, Chopra AK (2013). The exoribonuclease polynucleotide phosphorylase influences the virulence and stress responses of yersiniae and many other pathogens. Front. Cell. Infect. Microbiol..

[35] Sesto N (2014). A PNPase dependent CRISPR system in *Listeria*. PLoS Genet..

[36] Rouf SF (2011). Opposing contributions of polynucleotide phosphorylase and the membrane protein Nlpi to biofilm formation by *Salmonella enterica* serovar Typhimurium. J. Bacteriol..

[37] Saramago M, Domingues S, Viegas SC, Arraiano CM (2014). Biofilm formation and antibiotic resistance in *Salmonella* Typhimurium are affected by different ribonucleases. J. Microbiol. Biotechnol..

[38] Pobre V, Arraiano CM (2015). Next generation sequencing analysis reveals that the ribonucleases RNase II, RNase R and PNPase affect bacterial motility and biofilm formation in *E. coli*. BMC Genomics.

[39] Carzaniga T, Antoniani D, Dehò G, Briani F, Landini P (2012). The RNA processing enzyme polynucleotide phosphorylase negatively controls biofilm formation by repressing poly-N-acetylglucosamine (PNAG) production in *Escherichia coli* C. BMC Microbiol..

[40] Kühbacher, A., Cossart, P. & Pizarro-Cerdá, J. Internalization Assays for *Listeria monocytogenes*. in *Listeria monocytogenes—Methods and Protocols* (eds Jordan, K., Fox, E. M. & Wagner, M.) Vol. 1157, 167–178 (Springer New York, 2014). 10.1007/978-1-4939-0703-8_14 24792557

[41] Josenhans C, Suerbaum S (2002). The role of motility as a virulence factor in bacteria. Int. J. Med. Microbiol..

[42] Yang DC, Blair KM, Salama NR (2016). Staying in shape: the impact of cell shape on bacterial survival in diverse environments. Microbiol. Mol. Biol. Rev..

[43] Serra DO, Richter AM, Klauck G, Mika F, Hengge R (2013). Microanatomy at cellular resolution and spatial order of physiological differentiation in a bacterial biofilm. MBio.

[44] Martín‐Rodríguez AJ (2021). Regulation of colony morphology and biofilm formation in *Shewanella algae*. Microb. Biotechnol..

[45] Pinto SN (2019). The mechanism of action of pepR, a viral-derived peptide, against *Staphylococcus aureus* biofilms. J. Antimicrob. Chemother..

[46] Colagiorgi A, Di Ciccio P, Zanardi E, Ghidini S, Ianieri A (2016). A look inside the *Listeria monocytogenes* biofilms extracellular matrix. Microorganisms.

[47] Loza-Correa M (2019). The peptidoglycan and biofilm matrix of *Staphylococcus epidermidis* undergo structural changes when exposed to human platelets. PLoS ONE.

[48] Skogman ME, Vuorela PM, Fallarero A (2012). Combining biofilm matrix measurements with biomass and viability assays in susceptibility assessments of antimicrobials against *Staphylococcus aureus* biofilms. J. Antibiot..

[49] Borucki MK, Peppin JD, White D, Loge F, Call DR (2003). Variation in biofilm formation among strains of *Listeria monocytogenes*. Appl. Environ. Microbiol..

[50] Bertolesi GE, de Cidre LL, Stockert JC (1995). Formation and microscopical application of a fluorescent 1,10-phenanthrolinederivative of ruthenium red. Acta Histochem..

[51] Allerberger F, Wagner M (2010). Listeriosis: a resurgent foodborne infection. Clin. Microbiol. Infect..

[52] Temple ME, Nahata MC (2000). Treatment of listeriosis. Ann. Pharmacother..

[53] Jaishankar J, Srivastava P (2017). Molecular basis of stationary phase survival and applications. Front. Microbiol..

[54] Poyart C, Trieu-Cuot P (1997). A broad-host-range mobilizable shuttle vector for the construction of transcriptional fusions to beta-galactosidase in gram-positive bacteria. FEMS Microbiol. Lett..

[55] Autret N, Raynaud C, Dubail I, Berche P, Charbit A (2003). Identification of the *agr* locus of *Listeria monocytogenes*: role in bacterial virulence. Infect. Immun..

[56] Riedel CU (2009). AgrD-dependent quorum sensing affects biofilm formation, invasion, virulence and global gene expression profiles in *Listeria monocytogenes*. Mol. Microbiol..

[57] Postma PW, Lengeler JW, Jacobson GR (1993). Phosphoenolpyruvate:carbohydrate phosphotransferase systems of bacteria. Microbiol. Rev..

[58] Jeckelmann JM, Erni B (2020). The mannose phosphotransferase system (Man-PTS)—mannose transporter and receptor for bacteriocins and bacteriophages. Biochim. Biophys. Acta Biomembr..

[59] Erni B (2013). The bacterial phosphoenolpyruvate: sugar phosphotransferase system (PTS): an interface between energy and signal transduction. J. Iran. Chem. Soc..

[60] Allan RN (2014). Pronounced metabolic changes in adaptation to biofilm growth by *Streptococcus pneumoniae*. PLoS ONE.

[61] Suriyanarayanan T (2018). Quantitative proteomics of strong and weak biofilm formers of *Enterococcus faecalis* reveals novel regulators of biofilm formation. Mol. Cell. Proteom..

[62] Gopal S (2010). Maltose and maltodextrin utilization by *Listeria monocytogenes* depend on an inducible ABC transporter which is repressed by glucose. PLoS ONE.

[63] Deutscher, J. et al. Carbohydrate utilization by *Listeria monocytogenes* and its influence on virulence gene expression. in *Listeria Monocytogenes: Food Sources, Prevalence and Management Strategies* (ed. Hambrick, E. C.) 49–76 (Nova Science Pub Inc, 2014).

[64] Koomen J (2018). Gene profiling-based phenotyping for identification of cellular parameters that contribute to fitness, stress-tolerance and virulence of *Listeria monocytogenes* variants. Int. J. Food Microbiol..

[65] Noothalapati Venkata HN, Nomura N, Shigeto S (2011). Leucine pools in *Escherichia coli* biofilm discovered by Raman imaging. J. Raman Spectrosc..

[66] Valle J (2008). The amino acid valine is secreted in continuous-flow bacterial biofilms. J. Bacteriol..

[67] Bernier SP, Ha D-G, Khan W, Merritt JH, O’Toole GA (2011). Modulation of *Pseudomonas aeruginosa* surface-associated group behaviors by individual amino acids through c-di-GMP signaling. Res. Microbiol..

[68] Liu Z (2021). Integration of transcriptome and metabolome reveals the genes and metabolites involved in *Bifidobacterium bifidum* biofilm formation. Int. J. Mol. Sci..

[69] Huang Y (2013). Mutations in *gltB* and *gltC* reduce oxidative stress tolerance and biofilm formation in *Listeria monocytogenes* 4b G. Int. J. Food Microbiol..

[70] Kimura T, Kobayashi K (2020). Role of glutamate synthase in biofilm formation by *Bacillus subtilis*. J. Bacteriol..

[71] Wang Y (2012). Comparative proteomic analysis of *Streptococcus suis* biofilms and planktonic cells that identified biofilm infection-related immunogenic proteins. PLoS ONE.

[72] Wang Y (2019). pdh modulate virulence through reducing stress tolerance and biofilm formation of *Streptococcus suis* serotype 2. Virulence.

[73] Andrade JM, Pobre V, Matos AM, Arraiano CM (2012). The crucial role of PNPase in the degradation of small RNAs that are not associated with Hfq. RNA.

[74] Quendera AP (2020). RNA-binding proteins driving the regulatory activity of small non-coding RNAs in bacteria. Front. Mol. Biosci..

[75] Marujo PE (2000). RNase II removes the oligo(A) tails that destabilize the rpsO mRNA of *Escherichia coli*. RNA.

[76] Mengaud J (1991). Pleiotropic control of *Listeria monocytogenes* virulence factors by a gene that is autoregulated. Mol. Microbiol..

[77] Luo Q (2013). PrfA led to reduced biofilm formation and contributed to altered gene expression patterns in biofilm-forming *Listeria monocytogenes*. Curr. Microbiol..

[78] Janež N, Škrlj B, Sterniša M, Klančnik A, Sabotič J (2021). The role of the *Listeria monocytogenes* surfactome in biofilm formation. Microb. Biotechnol..

[79] Cámara M (2022). Economic significance of biofilms: a multidisciplinary and cross-sectoral challenge. NPJ Biofilms Microbiomes.

[80] Martínez LC, Vadyvaloo V (2014). Mechanisms of post-transcriptional gene regulation in bacterial biofilms. Front. Cell. Infect. Microbiol..

[81] Oliva G, Sahr T, Buchrieser C (2015). Small RNAs, 5’ UTR elements and RNA-binding proteins in intracellular bacteria: impact on metabolism and virulence. FEMS Microbiol. Rev..

[82] Christopoulou N, Granneman S (2022). The role of RNA‐binding proteins in mediating adaptive responses in Gram‐positive bacteria. FEBS J..

[83] Heydorn A (2000). Quantification of biofilm structures by the novel computer program COMSTAT. Microbiology.

[84] Combrouse T (2013). Quantification of the extracellular matrix of the *Listeria monocytogenes* biofilms of different phylogenic lineages with optimization of culture conditions. J. Appl. Microbiol..

[85] Dubois M, Gilles KA, Hamilton JK, Rebers PA, Smith F (1956). Colorimetric method for determination of sugars and related substances. Anal. Chem..

[86] Nakamura H, Takakura KI, Sone Y, Itano Y, Nishikawa Y (2013). Biofilm formation and resistance to benzalkonium chloride in *Listeria monocytogenes* isolated from a fish processing plant. J. Food Prot..

[87] Bradford MM (1976). A rapid and sensitive method for the quantitation of microgram quantities of protein utilizing the principle of protein-dye binding. Anal. Biochem..

[88] Sambrook J, Russell DW (2006). Purification of nucleic acids by extraction with phenol:chloroform. Cold Spring Harb. Protoc..

[89] Pobre V, Arraiano CM (2018). Characterizing the role of exoribonucleases in the control of microbial gene expression: differential RNA-Seq. Methods Enzymol..

[90] Lauer P, Chow MYN, Loessner MJ, Portnoy DA, Calendar R (2002). Construction, characterization, and use of two *Listeria monocytogenes* site-specific phage integration vectors. J. Bacteriol..

