# Supplementary material for: The ribonuclease PNPase is a key regulator of biofilm formation in Listeria monocytogenes and affects invasion of host cells
Source: NPJ Biofilms Microbiomes. 2023 Jun 7;9:34. doi: 10.1038/s41522-023-00397-1 (PMC10247797; doi:10.1038/s41522-023-00397-1)
Supplement: Supplementary file 1 — Supplemental Information [file 41522_2023_397_MOESM1_ESM.pdf]

Supplementary Information

Supplementary Table 1. RNA-seq analysis and DEG list. Tables provided in an excel file.

Supplementary Table 2. List of bacterial strains and plasmids used in this work.

Strains or plasmids	Description	References or Source
**Strains**		
Listeria monocytogenes		
EGDe (WT)	Wild-type strain, lineage II, serovar 1/2a	35
Δ*pnpA*	Wild-type derived strain carrying a null mutant of PNPase/Lmo1331	35
Δ*pnpA::pnpA*	Δ*pnpA* strain with integration of the *pnpA* gene in the genome	35
WT-P*lmo0048-lacZ*	Wild-type strain containing plasmid pTCV-P*lmo0048-lacZ*	This study
WT-P*lmo2006-lacZ*	Wild-type strain containing plasmid pTCV-P*lmo2006-lacZ*	This study
Δ*pnpA*-P*lmo0048-lacZ*	Δ*pnpA* strain containing plasmid pTCV-P*lmo0048-lacZ*	This study
Δ*pnpA*-P*lmo2006-lacZ*	Δ*pnpA* strain containing plasmid pTCV-P*lmo2006-lacZ*	This study
Escherichia coli		
DH5α	Strain used for cloning	Lab's stock
S17-1	Donor for plasmid conjugation	Lab's stock
**Plasmids**		
pTCV-*lacZ*	Kan^R; Transcriptional *lacZ* fusion vector; low copy-number	54
pTCV-P*lmo0048-lacZ*	Kan^R; Truncated *lmo0048* region (-174 to +1) inserted in pTCV-*lacZ* upstream of *lacZ*	This study
pTCV-P*lmo2006-lacZ*	Kan^R; Truncated *lmo2006* region (-117 to +1) inserted in pTCV-*lacZ* upstream of *lacZ*	This study

Supplementary Table 3. List of oligonucleotides used in this work.

Name	Sequence 5'-3'
**qPCR primers**	
GyrA-FWD	TGCTGGTGTTCGTGGTATTAG
GyrA-REV	ATTACGAAGCGGATATTGGGAG
lmo0048-FWD	GACCGCTGGAAAGATGATGAA
lmo0048-REV	CGATACCGTATACGAGAGCAAAC
lmo0096-FWD	AGATCCACGTTTCGGCAATA
lmo0096-REV	CGATTTCTACGCCACCTTCA
lmo0343-FWD	AAAGCAGTCCATACCCTCAC
lmo0343-REV	GTCTTCCTAAGAACGGGCTAAT
lmo0345-FWD	TTCGCGCAGCACAAATTC
lmo0345-REV	GGCGCCCATGGTCATAATA
lmo0784-FWD	ATTTGGCGGCAGTCCTTAT
lmo0784-REV	CCGCATAGAGAGCGTTTCA
lmo1052-FWD	GTCTAGGTTTCTACGCTCCAAC
lmo1052-REV	TTGAGGCACATCACGGTATC
lmo1053-FWD	CAAATGGCTCGTATGCGTTAC
lmo1053-REV	GCGTGCATTTCTGGTGTATG
lmo1733-FWD	TACAAGCCGCTCGATGTATG
lmo1733-REV	CGTCGTTCCACTCAGGAATTA
lmo1986-FWD	AATCCTCCAAGCGATGTAGATG
lmo1986-REV	GATAGCGAATAGGGAAGGAACAG
lmo2124-FWD	GACGATCTGTGCGACTTCTTTA
lmo2124-REV	CCGCCCAAGGAAGTAAGAAA
lmo2125-FWD	CGCAACCAATGCCTAATATTCC
lmo2125-REV	GAACCAGAAGCTGCATCAAAC
**Primers for cloning**	
pPL2-pnpA-BamHI	TATGGATCCTGGTAGGTCACCGTCGTAAC
pPL2-pnpA-SalI	GGAAGTCGACAGCGAGAAATGGCTTACCAC
P0048-FWD-EcoRI	GACGAATTCGGGTTTTTAAGCCTATTATACAG
P0048-REV-BamHI	CATGGATCCCTAATTCACCTCCACTAATATTTTAC
P2006-FWD-EcoRI	CTTGAATTCGAGTCAAGAACCTTGCAATAATTC
P2006-REV-BamHI	CTAGGATCCCTTCATCATCCTTTTGTTATATAAATTATC

pTCV-FWD	GTTGAATAACACTTATTCCTATC
pTCV-REV	CTTCCACAGTAGTTCACCACC
**Northern blot probes**	
lmo0048	GACAGATGCACTGTCACTGTTTG
lmo0096	GTGAGTTGCGAGGATAATTCCTA
lmo2006	GACTGGATCTCCTTCTGCAGTTGCGG
lmo2125	GTCTTTGTAGCCTGCGTCAACGG
prfA	GGTCCCGTTCTCGCTAATACTCG
hly	GGCGTCTTAGGACTTGCAGGCGGAGATG
inlA	GTGAGACCGTGTCTGTTACATTCG
mogR	GATTCCACCGTGTTCTTCAACGGCTTG
tmRNA	GGAGGATCAGCTATGCTACTG

Supplementary Figure 1. Unprocessed images for Figure 2a.

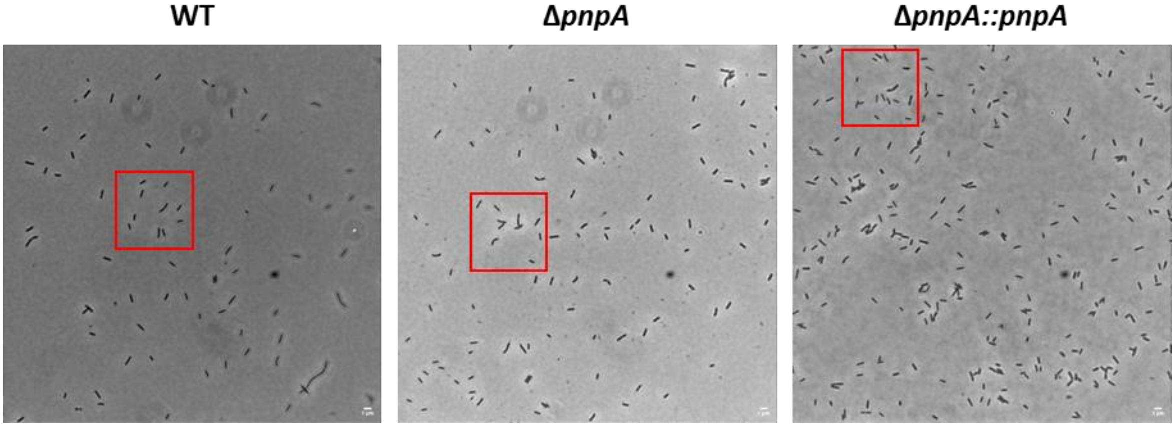

Rectangles indicate cropped areas.

Supplementary Figure 2. Unprocessed images for Figure 2c.

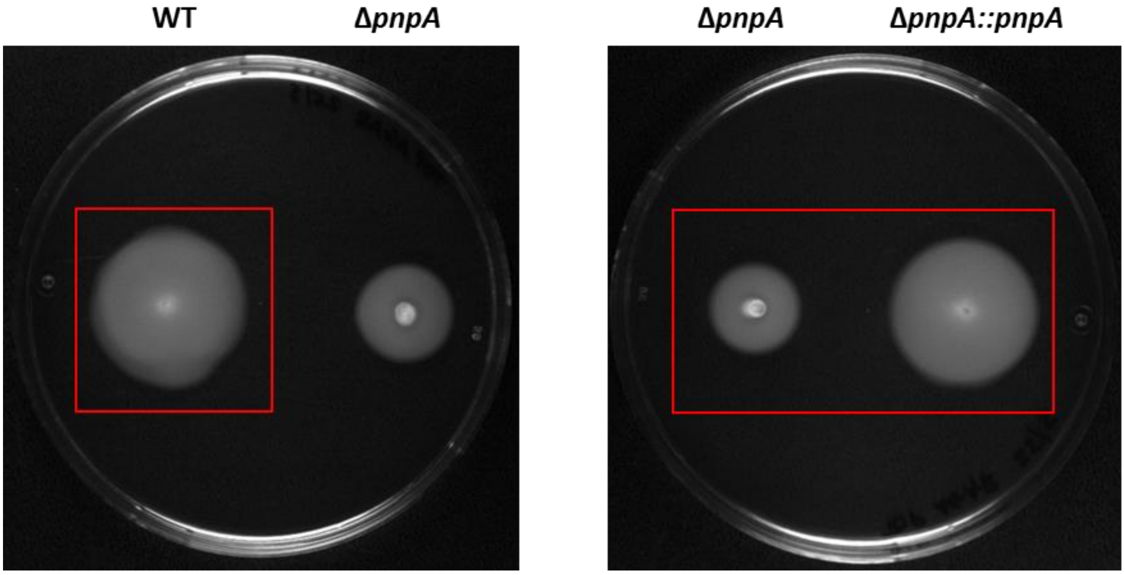

Rectangles indicate cropped areas, which were merged in a single image. Plates were inoculated in the same day and growth proceeded for the same period.

Supplementary Figure 3. Unprocessed images for Figure 7a.

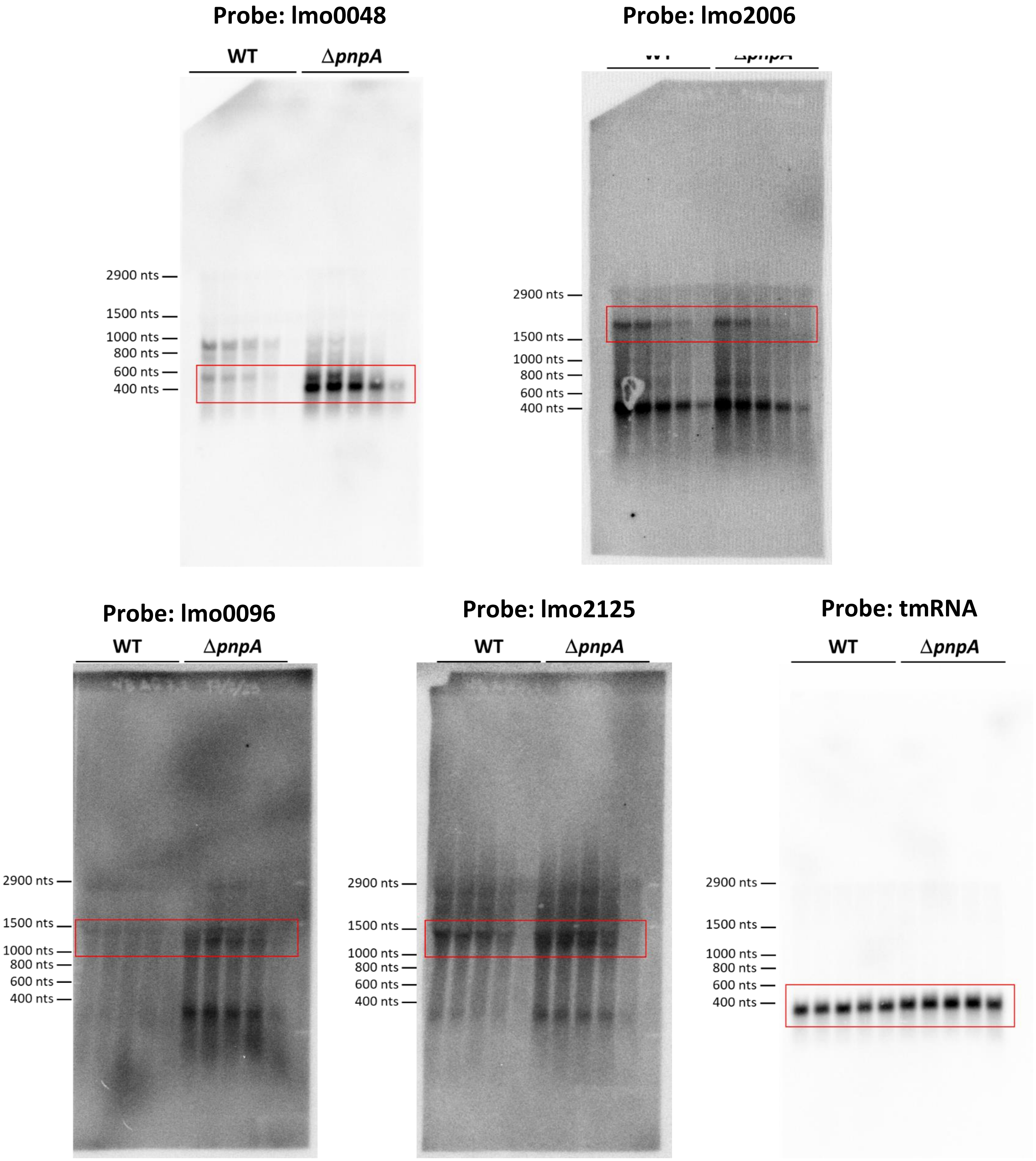

Rectangles indicate cropped areas.

Supplementary Figure 4. Unprocessed images for Figure 7c.

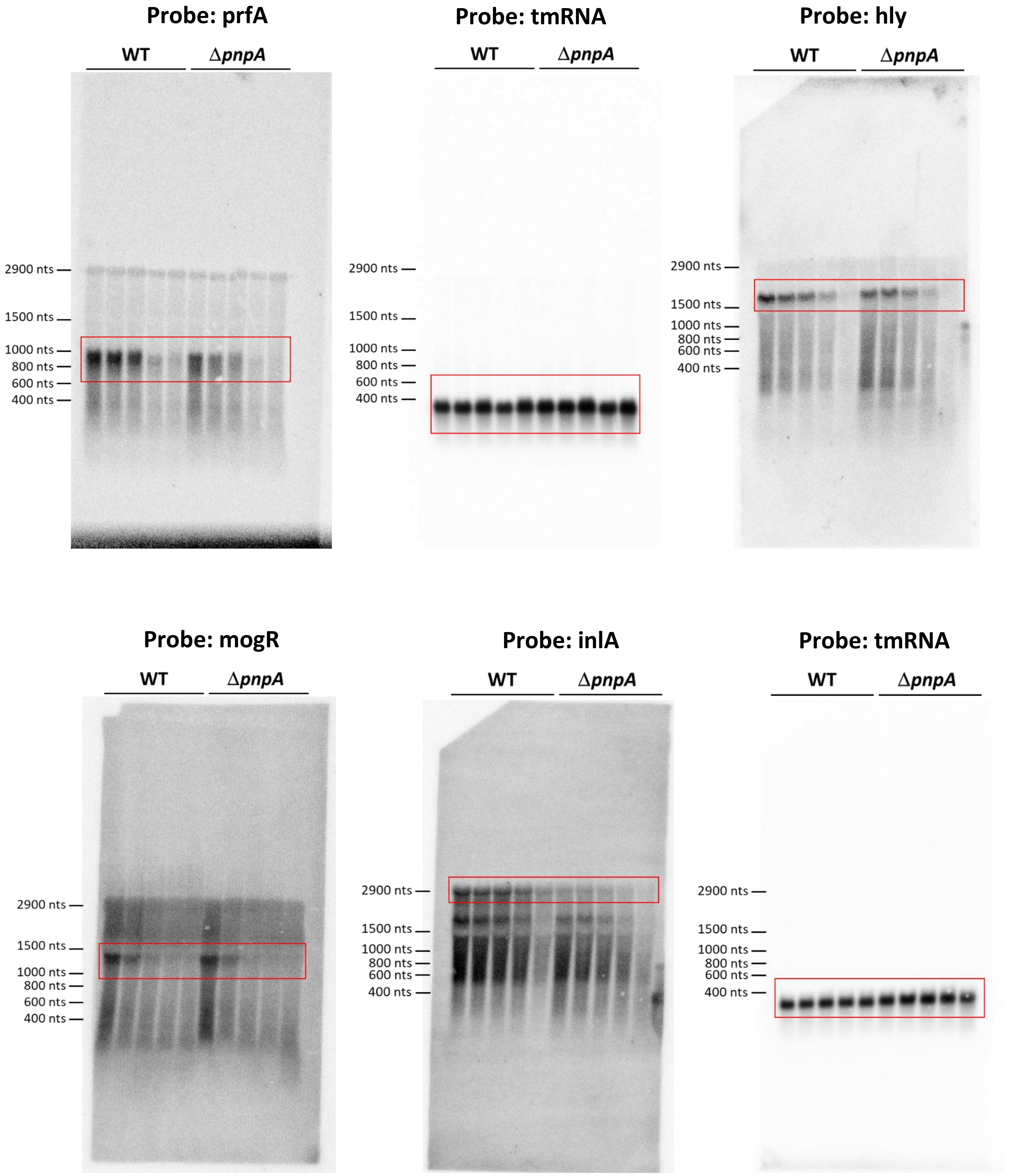

Rectangles indicate cropped areas.

Supplementary Figure 5. Unprocessed images for Figure 7d.

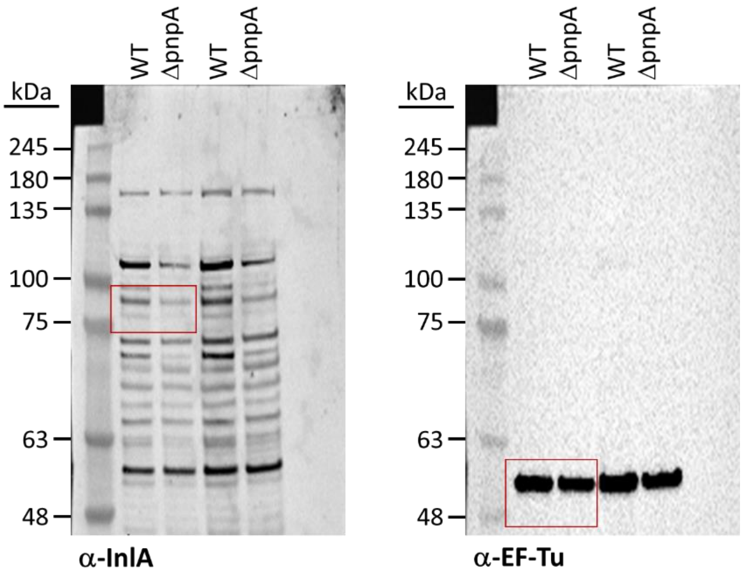

Rectangles indicate cropped areas.
