# Supplementary material for: The ribonuclease PNPase is a key regulator of biofilm formation in Listeria monocytogenes and affects invasion of host cells
Source: NPJ Biofilms Microbiomes. 2023 Jun 7;9:34. doi: 10.1038/s41522-023-00397-1 (PMC10247797; doi:10.1038/s41522-023-00397-1)
Supplement: Supplementary file 3 — supplementary table 1 [file 41522_2023_397_MOESM3_ESM.pdf]

Corresponding author(s): José Marques Andrade

Last updated by author(s): May 16, 2023

Reporting Summary

Nature Portfolio wishes to improve the reproducibility of the work that we publish. This form provides structure for consistency and transparency in reporting. For further information on Nature Portfolio policies, see our Editorial Policies and the Editorial Policy Checklist.

Statistics

For all statistical analyses, confirm that the following items are present in the figure legend, table legend, main text, or Methods section.

n/a	Confirmed	
☐	☒	The exact sample size (*n*) for each experimental group/condition, given as a discrete number and unit of measurement
☒	☐	A statement on whether measurements were taken from distinct samples or whether the same sample was measured repeatedly
☐	☒	The statistical test(s) used AND whether they are one- or two-sided<br>*Only common tests should be described solely by name; describe more complex techniques in the Methods section.*
☒	☐	A description of all covariates tested
☒	☐	A description of any assumptions or corrections, such as tests of normality and adjustment for multiple comparisons
☒	☐	A full description of the statistical parameters including central tendency (e.g. means) or other basic estimates (e.g. regression coefficient) AND variation (e.g. standard deviation) or associated estimates of uncertainty (e.g. confidence intervals)
☒	☐	For null hypothesis testing, the test statistic (e.g. *F*, *t*, *r*) with confidence intervals, effect sizes, degrees of freedom and *P* value noted<br>*Give P values as exact values whenever suitable.*
☒	☐	For Bayesian analysis, information on the choice of priors and Markov chain Monte Carlo settings
☒	☐	For hierarchical and complex designs, identification of the appropriate level for tests and full reporting of outcomes
☒	☐	Estimates of effect sizes (e.g. Cohen's *d*, Pearson's *r*), indicating how they were calculated

Our web collection on statistics for biologists contains articles on many of the points above.

Software and code

Policy information about availability of computer code

Data collection	No software was used
Data analysis	No software was used

For manuscripts utilizing custom algorithms or software that are central to the research but not yet described in published literature, software must be made available to editors and reviewers. We strongly encourage code deposition in a community repository (e.g. GitHub). See the Nature Portfolio guidelines for submitting code & software for further information.

Data

Policy information about availability of data

All manuscripts must include a data availability statement. This statement should provide the following information, where applicable:

- Accession codes, unique identifiers, or web links for publicly available datasets
- A description of any restrictions on data availability
- For clinical datasets or third party data, please ensure that the statement adheres to our policy

Transcriptomic dataset has been depoisted in NCBI's Gene Expression Omnibus and is publicly available through GEO Series accession number GSE210097

Research involving human participants, their data, or biological material

Policy information about studies with human participants or human data. See also policy information about sex, gender (identity/presentation), and sexual orientation and race, ethnicity and racism.

Reporting on sex and gender	n/a
Reporting on race, ethnicity, or other socially relevant groupings	n/a
Population characteristics	n/a
Recruitment	n/a
Ethics oversight	n/a

Note that full information on the approval of the study protocol must also be provided in the manuscript.

Field-specific reporting

Please select the one below that is the best fit for your research. If you are not sure, read the appropriate sections before making your selection.

☒ Life sciences ☐ Behavioural & social sciences ☐ Ecological, evolutionary & environmental sciences

For a reference copy of the document with all sections, see nature.com/documents/nr-reporting-summary-flat.pdf

Life sciences study design

All studies must disclose on these points even when the disclosure is negative.

Sample size	RNA-seq data and Northern blotting analysis were performed with RNA extracted from two independent biological replicates for each strain. Other experiments are the result of at least three independent biological samples, except Western blotting where two samples were analysed.
Data exclusions	Our RNA-seq data was sorted by statistical analysis. Gene features were considered as significantly up- or down-regulated in the mutant vs. WT samples using the following criteria: FDR<0.10, -1<log2FC<+1, log2CPM>3. Genes that do not follow these criiteria are not considered to be significant (i.e. no significant changes were detected between the strains in the same experimental condition). However, all data is made available in the NCBI's Gene Expression Omnibus.
Replication	We used an adjusted p-value correction (FDR) in the analysis of RNA-seq data. Whenever possible, data was statistically analysed to calculation of the SD or Se. A minimum of two independent experiments were performed in all assays, using different biological samples.
Randomization	This was not applied to our study as we work with bacterial cultures.
Blinding	Blinding was not possible as our experimental design implies that we always knmow what are the samples for the wild-type and the samples for the mutant strain.

Reporting for specific materials, systems and methods

We require information from authors about some types of materials, experimental systems and methods used in many studies. Here, indicate whether each material, system or method listed is relevant to your study. If you are not sure if a list item applies to your research, read the appropriate section before selecting a response.

Materials & experimental systems

n/a	Involved in the study	
☐	☒	Antibodies
☐	☒	Eukaryotic cell lines
☒	☐	Palaeontology and archaeology
☒	☐	Animals and other organisms
☒	☐	Clinical data
☒	☐	Dual use research of concern
☒	☐	Plants

Methods

n/a	Involved in the study	
☒	☐	ChIP-seq
☒	☐	Flow cytometry
☒	☐	MRI-based neuroimaging

Antibodies

Antibodies used	InlA antibody: Cusabio, CSB-PA758331HA01AAF, polyclonal<br>EF-Tu antibody: Abcam, ab90813, monoclonal
Validation	InlA antibody: recombinant Listeria monocytogenes serotype 4b Internalin-A protein<br>EF-Tu antibody: Anti-eEF1A1/EF-Tu antibody

Eukaryotic cell lines

Policy information about cell lines and Sex and Gender in Research

Cell line source(s)	HeLa ATCC® CCL-2™<br>HepG2 ATCC® HB-8065™
Authentication	None of the cell lines used were authenticated but they are well established cell lines and showed the typical microscopic phenotypes for each. In addition, these do not corresponding to the development of new cell lines.
Mycoplasma contamination	The cell lines were not specifically tested for mycoplasma contamination but microscopy and visual analysis of the cell cultures did not identify any mycoplasma contamination.
Commonly misidentified lines<br>(See ICLAC register)	n/a
